# Indication of Measures of Uncertainty for Statistical Significance in Abstracts of Published Oncology Trials: A Systematic Review and Meta-analysis

**DOI:** 10.1001/jamanetworkopen.2019.17530

**Published:** 2019-12-13

**Authors:** Samuel M. Rubinstein, Elizabeth A. Sigworth, Shervin Etemad, Richard L. Martin, Qingxia Chen, Jeremy L. Warner

**Affiliations:** 1 Division of Hematology and Oncology, Department of Medicine, Vanderbilt University, Nashville, Tennessee; 2 Department of Biostatistics, Vanderbilt University, Nashville, Tennessee; 3 Vanderbilt University School of Medicine, Nashville, Tennessee; 4 Department of Biomedical Informatics, Vanderbilt University, Nashville, Tennessee; 5 Vanderbilt-Ingram Cancer Center, Vanderbilt University Medical Center, Nashville, Tennessee

## Abstract

**Importance:**

There is growing consensus that reliance on *P* values, particularly a cutoff level of .05 for statistical significance, is a factor in the challenges in scientific reproducibility. Despite this consensus, publications describing clinical trial results with *P* values near .05 anecdotally use declarative statements that do not express uncertainty.

**Objectives:**

To quantify uncertainty expression in abstracts describing the results of cancer randomized clinical trials (RCTs) with *P* values between .01 and .10 and examine whether trial features are associated with uncertainty expression.

**Data Sources:**

A total of 5777 prospective trials indexed on HemOnc.org, as of September 15, 2019.

**Study Selection:**

Two-arm RCTs with a superiority end point with *P* values between .01 and .10.

**Data Extraction and Synthesis:**

Abstracts were evaluated based on an uncertainty expression algorithm. Ordinal logistic regression modeling with multiple imputation was performed to identify whether characteristics of study design, results, trial authors, and context *P* values were normalized by dividing by prespecified α value.

**Main Outcomes and Measures:**

Uncertainty expression in abstracts as determined by the algorithm and its association with trial and publication characteristics.

**Results:**

Of 5777 trials screened, 556 met analysis criteria. Of these, 222 trials (39.9%) did not express uncertainty, 161 trials (29.0%) expressed some uncertainty, and 173 trials (31.1%) expressed full uncertainty. In ordinal logistic regression with multiple imputation, trial features with statistically significant associations with uncertainty expression included later year of publication (odds ratio [OR], 1.70; 95% CI, 1.24-2.32; *P* < .001), normalized *P* value (OR, 1.36; 95% CI, 1.11-1.67; *P* = .003), noncooperative group studies (OR, 1.72; 95% CI, 1.12-2.63; *P* = .01), and reporting an end point other than overall survival (OR, 1.41; 95% CI, 1.01-1.96; *P* = .047). Funding source, number of authors, journal impact tier, author nationality, study of unapproved drugs, abstract word count, whether the marginal end point was a primary or coprimary end point, and effect size (in subgroup analysis) did not have statistically significant associations with uncertainty expression.

**Conclusions and Relevance:**

Published oncology articles with marginally significant results may often incompletely convey uncertainty. Although it appears that more uncertainty is expressed in recent abstracts, full uncertainty expression remains uncommon, and seemingly is less common when reporting overall survival, results with *P* values lower than α levels, and cooperative group studies.

## Key Points

**Question:**

Do the abstracts of oncology randomized clinical trials with results of marginal statistical significance express uncertainty, and what characteristics are associated with uncertainty expression?

**Findings:**

This systematic review and meta-analysis of 556 phase 3 randomized clinical trials found that most of the abstracts do not fully express uncertainty, and that uncertainty expression decreases when the *P* value surrounding a result is below its prespecified α level. The expression of uncertainty appears to be increasing over time.

**Meaning:**

It appears that a *P* value below the prespecified α level is often treated as a discrete threshold for statistical significance in publication of randomized clinical trials.

## Teaser

This systematic review and meta-analysis examines the expression of uncertainty in abstracts of published oncology randomized clinical trials.

## Introduction

Widely publicized clinical findings have often proven difficult to replicate in follow-up analyses.^1,2^ One factor is the high type I error rate associated with the ubiquitous threshold for statistical significance, which is a *P* value less than .05. Owing in part to concerns that this threshold has raised reproducibility concerns, the American Statistical Association released a consensus statement advising caution in interpretation of *P* values for hypothesis testing, emphasizing that *P* values do not measure the effect size of a result, nor do they inform the likelihood that the null hypothesis is false.^3^ However, it is common for publications to imply that a *P* value offers information on the probability of the null hypothesis (that a therapeutic intervention has no efficacy); instead, a *P* value provides the probability of observing the treatment effect assuming that the null hypothesis is valid.^4^ In reality, a *P* value of .05 continues to serve as a discrete threshold for statistical significance.^5^ The .05 threshold is so widely accepted as a threshold for publishable results that many collect data only until their results cross this threshold—a phenomenon known as “*P* hacking.”^6^ In response to this controversy, some have advocated for lowering the threshold by an order of magnitude^7^ or have questioned the validity of the concept of statistical significance.^8^ Others have emphasized inclusion of a confidence interval in clinical trial reporting to highlight the uncertainty of the results.^9^

There also remains substantial room for improvement in the rigor that is applied to reporting of what are considered to be positive clinical trial results.^1^ The magnitude of benefit reported as being clinically relevant declined between 1980 and 2010 in the reporting of clinical trials for non–small cell lung cancer, while at the same time reliance on surrogate end points increased.^10^ An analysis of randomized clinical trials (RCTs) with statistically nonsignificant results (ie, results with a *P* value >.05) reported that the results were presented with “spin” (misrepresentation of study findings) in a significant number of trials; as such, findings were reported in a manner that was inconsistent with the results.^9^ Spin has been shown to be associated with clinicians’ interpretation of trial results.^11^ This style of reporting has implications for the practice of medical oncology in particular. Antineoplastic therapies generally have among the lowest therapeutic indices and highest costs in medicine, meaning that the adverse effects associated with these therapies can outweigh the benefits in many scenarios. If the data supporting a treatment’s efficacy are marginal, and therefore the benefit of a treatment is questionable, clinicians need to be aware of this uncertainty and convey it to patients before making an informed decision about treatment.

In this article, we describe a systematic evaluation of RCT abstracts in oncology with a marginal *P* value result, which we define as between .01 and .10, to center our analysis on results near the widely accepted threshold of .05.^12,13^ We focused on whether uncertainty is expressed in the abstracts of published articles and examined factors potentially associated with uncertainty expression. We also examined the degree to which trials reporting these marginally significant results in the oncology literature expressed uncertainty and whether any secondary factors were associated with uncertainty expression.

## Methods

### Data Source

We derived our list of RCTs from HemOnc.org, a collaborative web-based knowledge base that aims to be a central repository of information regarding chemotherapy drugs and regimens that are used or have been used in routine cancer care.^14^ HemOnc.org has been continuously updated since 2011 and is the largest publicly available online repository of chemotherapy regimen information. Regimens included on HemOnc.org are discovered primarily through review of guidelines and the primary literature. The process of building HemOnc.org included systematic review of all American Society of Clinical Oncology, European Society of Medical Oncology, and National Comprehensive Cancer Network guidelines through December 31, 2018; all Cochrane Database of Systematic Reviews labeled as cancer specific (n = 685); and all *Lancet*, *JAMA*, and *New England Journal of Medicine* tables of contents between 1946 and December 31, 2018. In addition, the citations of any included material are hand-searched for additional citations. All RCT publications have been manually reviewed for comparative efficacy of the primary and key secondary end points, and the findings have been labeled in a structured format on the HemOnc.org website.

This study followed the Preferred Reporting Items for Systematic Reviews and Meta-analyses (PRISMA) reporting guideline where applicable.^15^ Although this is a meta-analysis of the expression of uncertainty in the oncology literature, the outcome of interest for this study is not a clinical outcome but rather the published text of clinical trial reporting, such that there is no intervention and comparison under examination. Typical features of meta-analyses delineated by the PRISMA guidelines, including aggregate measures of a clinical outcome or summary metric and assessment of bias in primary outcomes of the studies examined, are outside the scope of this analysis.

Efficacy is labeled following the recommendations by Pocock and Ware^13^ to translate statistical findings into plain English. As uncertainty may be meaningfully lower for results with *P* values less than .01 than between .01 and .05, and many journals simply report *P* values as less than .01 rather than reporting the precise *P* value, these guidelines emphasize the importance of conveying the uncertainty of results with *P* values greater than .01.^12,13^ With this justification, a regimen that shows an overall survival (OS) benefit above its comparator with a *P* value between .01 and .05 is labeled on the site as “seems to have superior OS,” whereas a regimen that shows an OS benefit less than .01 is labeled as having “superior OS.” To our knowledge, there are no other publicly available data sources that have similar labeling.

Efficacy labeling on HemOnc.org is first based on the primary outcome as reported by the clinical trial publication. If the primary outcome is a surrogate outcome and is positive with a *P* value less than .05, the least surrogate secondary outcome with *P* value of .10 or less is labeled instead. If the primary outcome is negative (*P* > .05), the trial is labeled as negative even if a secondary outcome is reported as positive. Representative examples are reported in eTable 1 in the Supplement. In addition, end points are relabeled if an interim update is published with new findings, such as loss of statistical significance for a primary end point; in these cases, the label on HemOnc.org is marked with an asterisk and a note of the format “reported efficacy is based on the YYYY update” is added.

**Figure 1.**
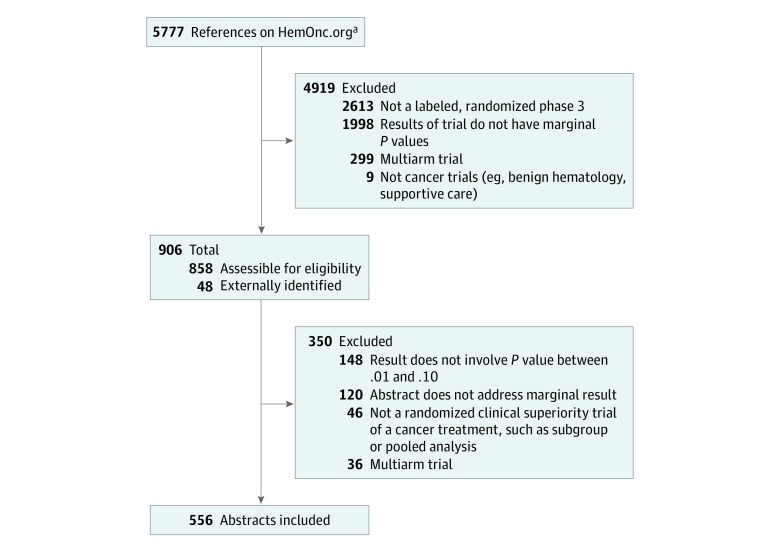
Selection of Abstracts for Analysis ^a^References included in the analysis are from published prospective trials with results only; abstract-only references, trial protocol descriptions, meta-analyses, reviews (systematic or otherwise), case reports, and case series were excluded.

In this study, a frozen version of the HemOnc.org text content from September 15, 2019, was programmatically screened for 2-arm RCTs with a seems to have label or an asterisk indicating an interim update to efficacy. This was a 3-step process (Figure 1). The first step used an automated-parsing algorithm to exclude trials not suitable for this analysis, such as noncancer trials; the second involved a manual review of candidate abstracts for eligibility; and the third involved a manual screen of regimens with complicated comparative designs not parsed in step 1. Trials that involved more than 2 arms, with or without factorial designs, were excluded from the analysis, as the primary discussions of such articles often do not focus on the specific result that is statistically marginal. The specific statement in the abstract results section that reported the statistically marginal result, as well as the concluding statement of the abstract, were analyzed for expression of uncertainty. Abstracts of updates to clinical trials were included in this analysis. In some cases, the category of statistical significance for the initial and updated analyses differed; the process for selecting which abstract to include in the analysis is summarized in eTable 2 in the Supplement.

### Uncertainty Scoring Algorithm

**Figure 2.**
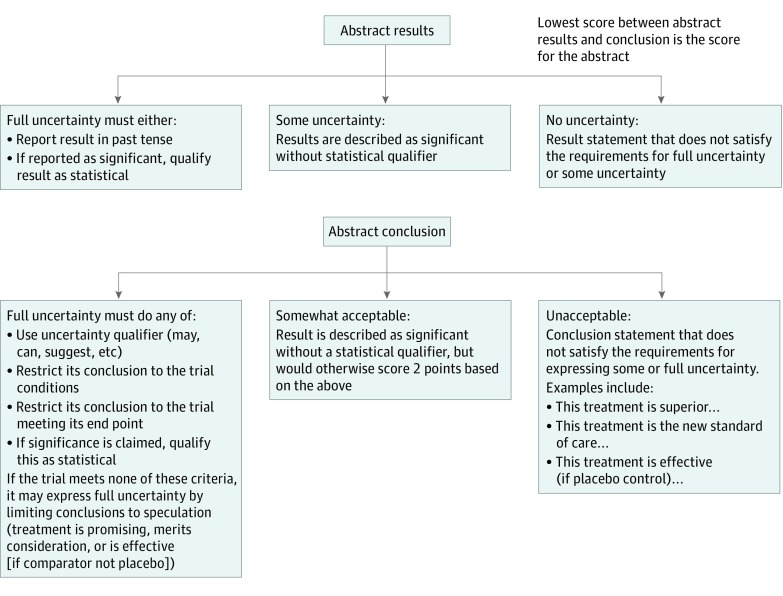
Algorithmic Approach for Evaluation of Uncertainty Graphical representation of the algorithm used to systematically evaluate the abstracts for uncertainty. A rationale is provided in the eAppendix in the Supplement.

To our knowledge, no consensus guidelines currently exist for best practices in uncertainty expression in the medical literature, although the imperative for increased uncertainty expression in the medical literature has been recognized.^16^ We therefore have proposed and developed a systematic approach for evaluating uncertainty expression based on 3 factors: whether reporting is restricted to the conditions of the trial, whether speculative language was used, and whether the significance of results is qualified as statistical (Figure 2). The logic behind the algorithm is described more fully in the eAppendix in the Supplement. We applied this algorithm to evaluate the abstracts for this study. The same approach was applied whether the marginal end point was OS or a surrogate of OS.

To assess interrater variability, after the primary abstractor (S.M.R.) reviewed the entire data set, the algorithm was given to a second abstractor (R.L.M.) along with a 50-abstract tutorial set with answers provided. Following review of this material, the second abstractor was given an additional set of 50 trials to review and evaluate based on that individual’s understanding of the algorithm. Interrater reliability was assessed using Fleiss κ and was computed using the R irr package, version 0.84.1 (R Foundation).^17,18^

### Data Collected for Analysis

The following metadata for each included RCT were recorded for analysis: (1) year of publication; (2) whether a for-profit pharmaceutical company funded the study; (3) the journal impact tier of publication (eTable 3 in the Supplement); (4) the total number of authors; (5) a score of 95% CI expression (eTable 4 in the Supplement); (6) the *P* value of the marginal result divided by prespecified α level; (7) the nationality of the authors’ affiliations (United States, non–United States, or collaborations between US and non-US authors); (8) whether the studies included a drug that, at the time of the RCT publication, was unapproved by the US Food and Drug Administration (FDA) or similar international regulatory bodies; (9) whether the study was conducted by a non–industry-funded cooperative group; (10) where reported, a point estimate for hazard ratio concerning the marginal end point; (11) the 95% CI surrounding the hazard ratio; (12) whether the marginal end point was a prespecified primary or coprimary end point; (13) whether the marginal end point was OS or a surrogate end point; and (14) abstract word count.

We examined results reporting for the presence of a 95% CI around the effect size to examine whether 95% CI expression was associated with uncertainty expression and scored articles on the degree to which a 95% CI was included (eTable 4 in the Supplement). Funding source information was extracted from the body of the article when available. If this information was not present in the body of the article, the metadata were searched, as well as the ClinicalTrials.gov record, when available.

Data regarding whether a trial involved an unapproved drug and whether a trial was conducted by a cooperative group are contained within the HemOnc.org ontologic framework and were abstracted from this database programmatically.^19^ The FDA approval dates of all drugs included on HemOnc.org as well as the publication dates are concepts within the ontologic framework. If the FDA approval date was after or the same year as the RCT publication (as in the latter case, the trial must have been designed and conducted before approval), the trial was determined to have been conducted before FDA approval of all drugs. Trials that contained unapproved drugs were manually screened for approval by international agencies and, if so, the first date of international approval was used.

### Statistical Analysis

The outcome of interest was the ordinal uncertainty expression as evaluated by the 3-level algorithm (full uncertainty, some uncertainty, no uncertainty). Continuous variables, such as year and number of authors, were summarized as median and interquartile range as well as mean (SD) for each level of outcome variable and compared using the Kruskal-Wallis test.^20^ Categorical variables were summarized as frequencies and counts. An ordinal logistic regression model or multivariable proportional odds model (eAppendix in the Supplement) was used to simultaneously study the associations of the covariates with uncertainty expression, allowing for nonlinear associations for year from 1974 and number of authors with restricted cubic spline functions with 5 knots.^21^ With 15 *df* per parameter in the model in complete case analysis, no variable selection procedure was implemented in the analysis. Multiple imputation with 150 imputations, assuming missingness at random for funding resource and journal tier, was used.^22^ Two-sided *P* values less than .05 were considered statistically significant. Ordinal logistic regression models and multiple imputation were performed with Hmisc and rms packages in R, version 3.4.4.^23,24^

Using the popower function from the Hmisc package, we analyzed our power to detect a range of odds ratios (ORs) given our current sample size, when binary predictors were in a 2:1 ratio (funding source, study group) or heavily imbalanced (10:1, whether experimental or control preferred). We found that in the 2:1 case, our current sample size gave us 80% power to detect an OR of 0.62 or lower (or ≥1.6 in the other direction); in the 10:1 imbalanced group case, we had 80% power to detect an OR of 0.48 or lower (or ≥2.08 in the other direction).

## Results

A total of 5777 articles were analyzed with a total of 3218 efficacy labels in the context of superiority trials (eTable 5 in the Supplement) Of these trials, 4919 were programmatically excluded, leaving 858 abstracts available for analysis. An additional 48 abstracts were identified external to the pruning algorithm for review, and in total, 906 were manually reviewed for inclusion in the analysis. Of these, 556 abstracts were suitable for inclusion (Figure 1). The PubMed identification numbers and titles of the 556 abstracts analyzed are reported in eTable 6 in the Supplement. The 2 abstractors of clinical trial uncertainty (S.M.R., R.L.M.) gave the same assessment on 45 of 50 (90.0%) mutually assessed abstracts (Fleiss κ, 0.85).

Characteristics of the included abstracts are reported in eTable 5 in the Supplement. Median year of publication of the abstracts was 2009 (range, 1974-2019). The median number of authors was 15 (range, 2-54). Three-hundred thirty-two abstracts (59.7%) had exclusively non-US authors, 96 abstracts (17.3%) had exclusively US authors, and 128 abstracts (23.0%) were of trials with collaborations between US and non-US authors. One hundred ninety-one trials (34.4%) were funded exclusively by grants, and 286 trials (51.4%) were at least partially funded by industry; funding source was missing for 79 (14.2%) of the RCTs. Of the 556 abstracts evaluated, 222 reported trials (39.9%) that did not express uncertainty, 161 abstracts (29.0%) expressed some uncertainty, and 173 abstracts (31.1%) expressed full uncertainty.

**Figure 3.**
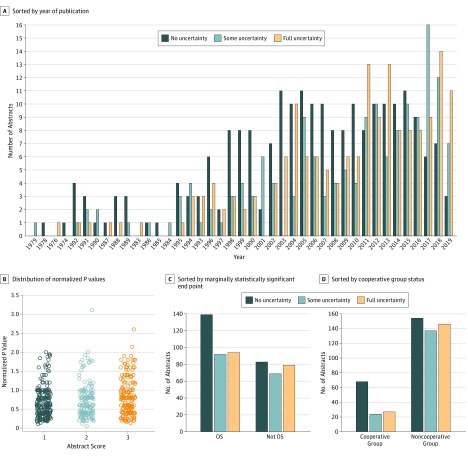
Abstract Uncertainty Expression by Year of Publication, Normalized *P* Values, Marginally Statistically Significant End Point, and Cooperative Group Status A, Abstract uncertainty expression sorted by year of publication. B, Distribution of normalized *P* values for abstracts with each degree of uncertainty expression. C, Abstract uncertainty expression sorted by the marginally statistically significant end point (overall survival [OS], or a surrogate of OS). D, Abstract uncertainty expression sorted by cooperative group status.

**Figure 4.**
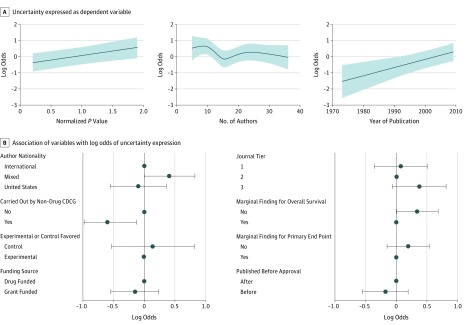
Multivariable Regression of Uncertainty Expression on Trial Predictors Multivariable logistic regression of uncertainty expression on various trial covariates. These figures show estimated values of the log odds at each value of the continuous factor while assuming all other variables are at their set reference levels (either prespecified for categorical variables or taken as the median for continuous variables). A, Multivariable logistic regressions with uncertainty expression as the dependent variable; the left side demonstrates the association between normalized *P* value and uncertainty expression, the middle examines the association between number of authors and uncertainty expression (not statistically significant), and the right side demonstrates the association between year of publication and uncertainty expression (statistically significant). B, Association of ordinal and categorial variables with the log odds of uncertainty expression. CDCG indicates Company-Driven Cooperative Group; error bars, 95% CIs for the estimate of the log odds.

Trial features with statistically significant associations with uncertainty expression after multiple imputation are shown in Figure 3: later year of publication (OR, 1.70; 95% CI, 1.24-2.32; *P* < .001), lower normalized *P* value (OR, 1.36; 95% CI, 1.11-1.67; *P* = .003), noncooperative group studies (OR, 1.72; 95% CI, 1.12-2.63; *P* = .01), and reporting an end point other than OS (OR, 1.41; 95% CI, 1.01-1.96; *P* = .047). Funding source, publication before regulatory drug approval, author nationality, confidence interval expression score, abstract word count, whether the marginal end point was a coprimary end point, journal tier, and number of authors were not associated with uncertainty expression after multiple imputation. Regression results are shown in Figure 4 and eFigure 1 in the Supplement.

In the subgroup analysis to evaluate the association of effect size point estimate with uncertainty expression, there was greater uncertainty expression with lower hazard ratios, although this association was not statistically significant (OR, 1.22; 95% CI, 0.98-1.54; *P* = .08). As in the complete model, publication year, normalized *P* value, noncooperative studies, and abstracts reporting end points other than OS expressed greater uncertainty. In this subanalysis, author nationality had a statistically significant association with uncertainty expression, with collaborations between US and non-US authors expressing more uncertainty than abstracts written by only US authors (OR, 2.38; 95% CI 1.14-5.00; *P* = .02) and only non-US authors (OR, 2.08; 95% CI, 1.26-3.33; *P* = .003). The subgroup analysis is summarized in eFigure 2 and eFigure 3 in the Supplement.

## Discussion

Publication of RCT results is the primary means by which knowledge of new and potentially effective therapies is disseminated. A definitive trial result is usually a factor in some or all of the following: new drug approvals, uptake of new combination therapies by practicing clinicians, insurance coverage decisions for new therapies, and inclusion in guidelines and compendia. The phrasing of the findings has particular importance to many constituents: clinicians, approval agencies, pharmacy benefit managers, and, by extension, the patients who receive these therapies.

To our knowledge, this is the largest analysis of uncertainty phrasing in oncology RCTs with statistically marginal results to date. We found that nearly 70% of the abstracts presented findings in a way that could be construed as definitive, even when the reported *P* value suggests a substantial risk of a type I error. Our focus on abstracts was intentional, given that many clinicians rely on abstracts as the primary sources of information that may change their clinical practice, despite well-recognized limitations in fully conveying the results of a trial.^25,26^ It would appear that clinicians increasingly rely on third-party knowledge bases that are often then integrated into clinical decision support systems.^27,28^ If the literature is biased toward overly optimistic statistically inaccurate interpretation of results, this bias may be associated with the validity of the meta-analyses and knowledge summarization required to generate these products. Systematic biases in the phrasing of the literature curated by knowledge bases are likely to be associated with automated curation efforts, such as CancerLinQ and other learning health systems used in oncology.^29,30^

Our finding that uncertainty expression is far from universal in the oncology literature is in line with other analyses.^31,32^ However, unlike previous analyses, our algorithm demonstrated high interobserver correlation without the need for post hoc resolution of disagreement. The finding that uncertainty expression is improving over time may be associated with an increase in published position articles and awareness regarding the aforementioned reproducibility concerns. There was a statistically significant tendency for uncertainty expression to decrease when the *P* value of a result is below the α level. In subgroup analysis, normalized *P* values had a more statistically significant association with uncertainty expression than the effect size. This finding further supports the hypothesis that the oncology community treats a *P* value of .05 as a cutoff not only for publication but also for external validity of a result. We also found that marginal OS results were conveyed with less uncertainty than marginal results that are surrogates of OS. Owing to the high clinical relevance of OS as a trial end point, it is understandable that marginally positive OS results are conveyed with more enthusiasm, and therefore less uncertainty, than other marginal results. It is also possible that awareness of the limitations of surrogate end points is growing, resulting in increasing uncertainty expression for trials that only show superiority in a surrogate end point.^33,34^

In contrast to other published data on the topic of spin in the oncology literature, we did not find an association between funding source and uncertainty expression. This finding is somewhat at odds with the widely held view that pharmaceutical industry–funded trials are more likely to be biased.^35,36,37^ However, funding sources are often difficult to discern from published material, especially in older studies, so we advise interpreting these results with caution.

### Limitations

This analysis has several limitations. First, HemOnc.org aims to capture all oncology RCTs but is not complete and disproportionately includes RCTs that are published in very high-impact journals or endorsed by guidelines. Second, the algorithm proposed for evaluating uncertainty expression is imperfect. Although the interobserver variability is low, there remain cases in which 2 individuals may interpret uncertainty phrasing differently based on the rules described herein. Subtle messaging, such as the use of tone, is not considered. Existing technologies for programmatically parsing text, such as natural language processing, are not currently capable of reliably evaluating tone in technical documents, and thus an automated approach is not plausible. Better algorithms may be developed in the future based on collaborative efforts, and we hope that this work sparks a discussion within the community regarding best practices for uncertainty expression. Third, we did not evaluate uncertainty expression of results with *P* values below .01. Although our analysis focused on uncertainty expression for results that marginally cleared the widely accepted *P* value threshold, uncertainty should be expressed for the vast majority of scientific results, including those for which the *P* value crosses .01. Future analyses will focus on how descriptions of these less-marginal results express uncertainty. Fourth, we did not analyze multiarm or factorial trials owing to concerns with the generalizability of the algorithm to these trials; this analysis is a planned focus of future work. Fifth, we did not evaluate whether each drug included was approved for the specific indication of the study, as opposed to approved at all. Our focus on the first date of drug approval was intentional because off-label drug use is common in oncology, but there may remain incentives to underexpress uncertainty if a study is aimed at obtaining a new indication for an already approved drug, and this question may merit additional study.^38^ Sixth, we did not examine individual author’s conflict of interest disclosures, which may represent a source of indirect funding; given the increased focus on this issue in the scientific literature, this source of support will be a focus of future work.^38,39^

## Conclusions

The results suggest that clinical trials commonly fail to convey uncertainty when describing results of marginal statistical significance. These results are often conveyed as definitively demonstrating that the null hypothesis of an experiment is false, which may be associated with reliance by the oncology community on a discrete threshold for statistical significance. Many prominent voices in the scientific community are advocating for a change in this culture, and we believe our data support their conclusion.^40,41,42^

## Notes

Supplement. **eAppendix.** Rationale of the Algorithm for Describing Uncertainty Expression and Explanation of a Proportional Odds Model **eFigure 1.** Forest Plot for Correlates of Uncertainty Expression **eFigure 2.** Visualization of Model for Subgroup Analysis **eFigure 3.** Forest Plot for Correlates of Uncertainty Expression in Subgroup Analysis **eTable 1.** Example Labeling of Comparative Efficacy of Superiority Trials on HemOnc.org **eTable 2.** Inclusion of Marginal Initial and Subsequent Analyses **eTable 3.** Journal Tiers as Defined in This Analysis **eTable 4.** Scoring Algorithm for Confidence Intervals **eTable 5.** Efficacy Labels for Trials on HemOnc.org **eTable 6.** PMIDs and Titles of Examined Abstracts
