# Error in Abstract

**DOI:** 10.1001/jamanetworkopen.2019.21064

**Published:** 2020-01-17

In the Original Investigation titled “Indication of Measures of Uncertainty for Statistical Significance in Abstracts of Published Oncology Trials: A Systematic Review and Meta-analysis,”^1^ published December 13, 2019, there was a run-on sentence in the Abstract. The sentence presented as “Ordinal logistic regression modeling with multiple imputation was performed to identify whether characteristics of study design, results, trial authors, and context *P* values were normalized by dividing by prespecified α value” should have appeared as “Ordinal logistic regression modeling with multiple imputation was performed to identify whether characteristics of study design, results, trial authors, and context were associated with uncertainty expression. *P* values were normalized by dividing by prespecified α value.” This article has been corrected.^1^
