# Supplementary material for: Indication of Measures of Uncertainty for Statistical Significance in Abstracts of Published Oncology Trials: A Systematic Review and Meta-analysis
Source: JAMA Netw Open. 2019 Dec 13;2(12):e1917530. doi: 10.1001/jamanetworkopen.2019.17530 (PMC6991218; doi:10.1001/jamanetworkopen.2019.17530)
Supplement: Supplement. — eAppendix. Rationale of the Algorithm for Describing Uncertainty Expression and Explanation of a Proportional Odds Model eFigure 1. Forest Plot for Correlates of Uncertainty Expression eFigure 2. Visualization of Model for Subgroup Analysis eFigure 3. Forest Plot for Correlates of Uncertainty Expression in Subgroup Analysis eTable 1. Example Labeling of Comparative Efficacy of Superiority Trials on HemOnc.org eTable 2. Inclusion of Marginal Initial and Subsequent Analyses eTable 3. Journal Tiers as Defined in This Analysis eTable 4. Scoring Algorithm for Confidence Intervals eTable 5. Efficacy Labels for Trials on HemOnc.org eTable 6. PMIDs and Titles of Examined Abstracts [file jamanetwopen-2-e1917530-s001.pdf]

Supplementary Online Content

Rubinstein SM, Sigworth EA, Etemad S, Martin RL, Chen Q, Warner JL. Indication of measures of uncertainty for statistical significance in abstracts of published oncology trials: a systematic review and meta-analysis. *JAMA Netw Open*. 2019;2(12):e1917530. doi:10.1001/jamanetworkopen.2019.17530

eAppendix. Rationale of the Algorithm for Describing Uncertainty Expression and Explanation of a Proportional Odds Model

eFigure 1. Forest Plot for Correlates of Uncertainty Expression

eFigure 2. Visualization of Model for Subgroup Analysis

eFigure 3. Forest Plot for Correlates of Uncertainty Expression in Subgroup Analysis

eTable 1. Example Labeling of Comparative Efficacy of Superiority Trials on HemOnc.org

eTable 2. Inclusion of Marginal Initial and Subsequent Analyses

eTable 3. Journal Tiers as Defined in This Analysis

eTable 4. Scoring Algorithm for Confidence Intervals

eTable 5. Efficacy Labels for Trials on HemOnc.org

eTable 6. PMIDs and Titles of Examined Abstracts

This supplementary material has been provided by the authors to give readers additional information about their work.

© 2019 Rubinstein SM et al. *JAMA Network Open.*

eAppendix. Rationale of the Algorithm for Describing Uncertainty Expression and Explanation of a Proportional Odds Model

Rationale of the algorithm for describing uncertainty expression

Due to publication bias, the majority of trials that are published will be positive experiments, meaning that the pre-specified alpha for statistical significance will be crossed. It is reasonable for trials that report successful results to declare the trial as positive. The problem comes in generalizing these conclusions to non-trial conditions in a declarative manner. This may not be appropriate at any p-value, but is particularly important for marginal results. With this in mind, the following criteria were established:

1. "Full uncertainty expression": In order for a trial to fully express uncertainty, it should meet the following criteria:
    a. Make clear that the conclusion only applies to the condition of the trial. This can be satisfied in a number of ways. A trial can restrict reporting of the results, or drawing of conclusions, to the past tense (for example, "regimen A *was* associated with improved overall survival compared to regimen B"). This also includes abstracts that only draw conclusions on the conditions of the trial itself (for example, "this study is positive for its pre-specified endpoint for superiority of regimen A over regimen B") .
        i. If the abstract expands the conclusions to conditions outside of the trial, it must do so using speculative language (for example, "regimen A may be considered an option") or a word that directly conveys uncertainty (for example, "regimen A may be associated with improved overall survival compared to regimen B").
    b. If a conclusion comments on the "significance" of a result, it must qualify this as "statistical", as some trials will demonstrate a statistically significant, but clinically insignificant benefit.

© 2019 Rubinstein SM et al. *JAMA Network Open.*

2. “Some uncertainty expression”: Trials that constrain their reporting to the conditions of the trial successfully, but do not clearly identify whether the significance of a result is clinical or statistical in nature, are conveying some uncertainty in the result. Although problematic, many in the community use the phrases “significant” and “statistically significant” interchangeably, or may be pressed to do this due to abstract word count requirements. We therefore labeled trials that meet the first criteria for uncertainty expression, but not the second, as expressing “some uncertainty”.

3. "No uncertainty expression”: In order for an abstract to be scored as “expressing no uncertainty”, it had to both fail to make clear that the conclusions are restricted to the conditions of the study and fail to qualify the conclusions in a speculative capacity (for example, “regimen A is superior to regimen B”).

Explanation of a proportional odds model

The proportional odds model is used with an ordinal, discrete outcome (such as our categorization of uncertainty expression) to examine how various predictors affect the odds of falling into higher values of the outcome as opposed to lower values of the outcome. For example, consider the estimated odds ratio for publication year of 1.7. This means that for a one unit increase in year of publication, with all other variables constant, the odds of that abstract expressing full uncertainty are 1.7 times higher than it expressing some or no uncertainty. Likewise, the odds of that abstract expressing some or full uncertainty, as opposed to no uncertainty, are 1.7 times higher. This interpretation relies on the assumption that the relationship between each paired outcome group is consistent, i.e. that the coefficients describing the relationship between no uncertainty expression and some or full uncertainty expression are the same as those describing the relationship between no or some uncertainty expression and full uncertainty expression.

© 2019 Rubinstein SM et al. *JAMA Network Open.*

eFigure 1. Forest plot for correlates of uncertainty expression

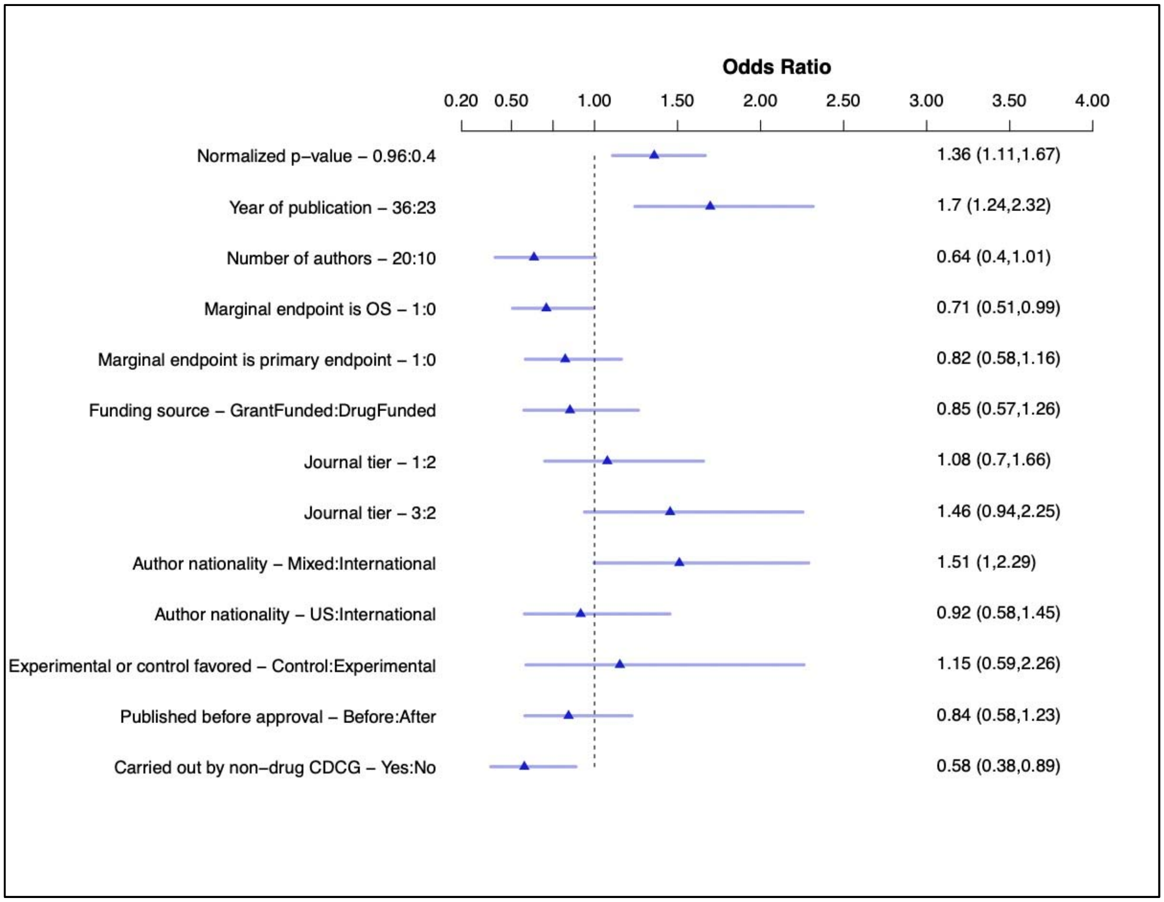

Forest plot demonstrating the magnitude of effect of each categorical covariate on uncertainty expression. Shaded error bars indicate the 95% CI's around each odds ratio graphically.

© 2019 Rubinstein SM et al. *JAMA Network Open.*

eFigure 2. Visualization of model for subgroup analysis

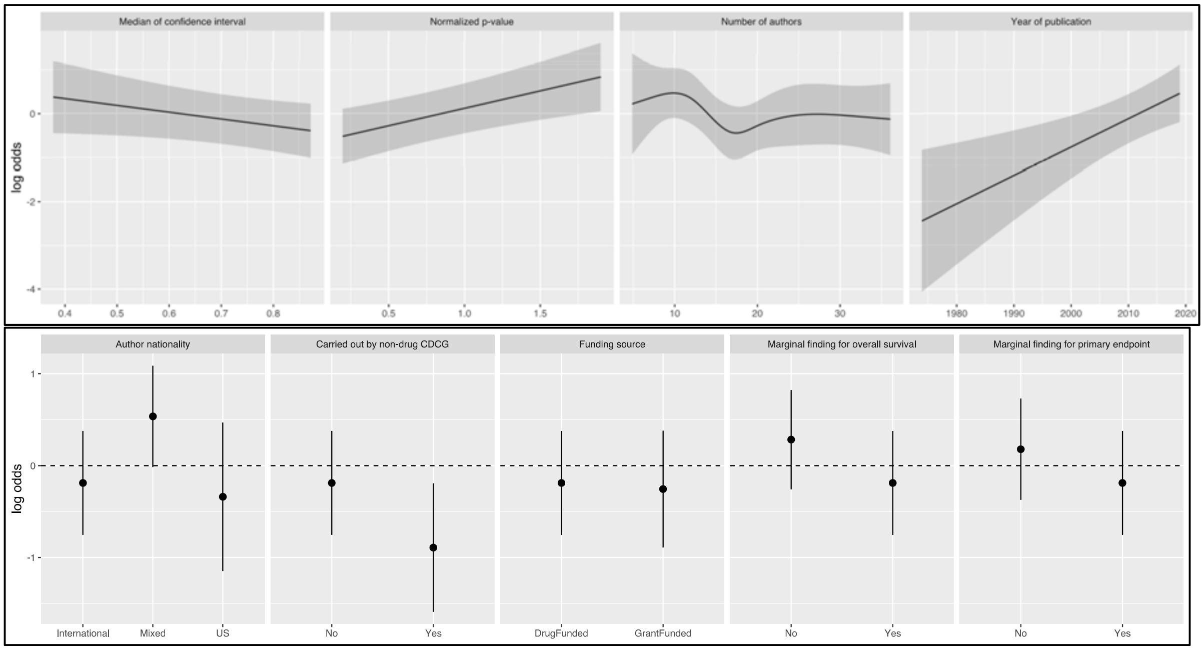

Multivariable logistic regression of uncertainty expression on various trial covariates in the subgroup of trials that expresses a point estimate for a hazard ratio for a time-dependent endpoint. The top panel is a graphical representation of multivariable logistic regressions with uncertainty expression as the dependent variable; from left to right, relationships with the point-estimate of hazard ratio (not statistically significant), normalized p-value (statistically significant), number of authors (not statistically significant) and year of publication (statistically significant). The x-axes for each show the values of each variable. The bottom panel demonstrates the relationship of ordinal and categorial variables with the log odds of uncertainty expression.

© 2019 Rubinstein SM et al. *JAMA Network Open.*

eFigure 3. Forest plot for correlates of uncertainty expression in subgroup analysis

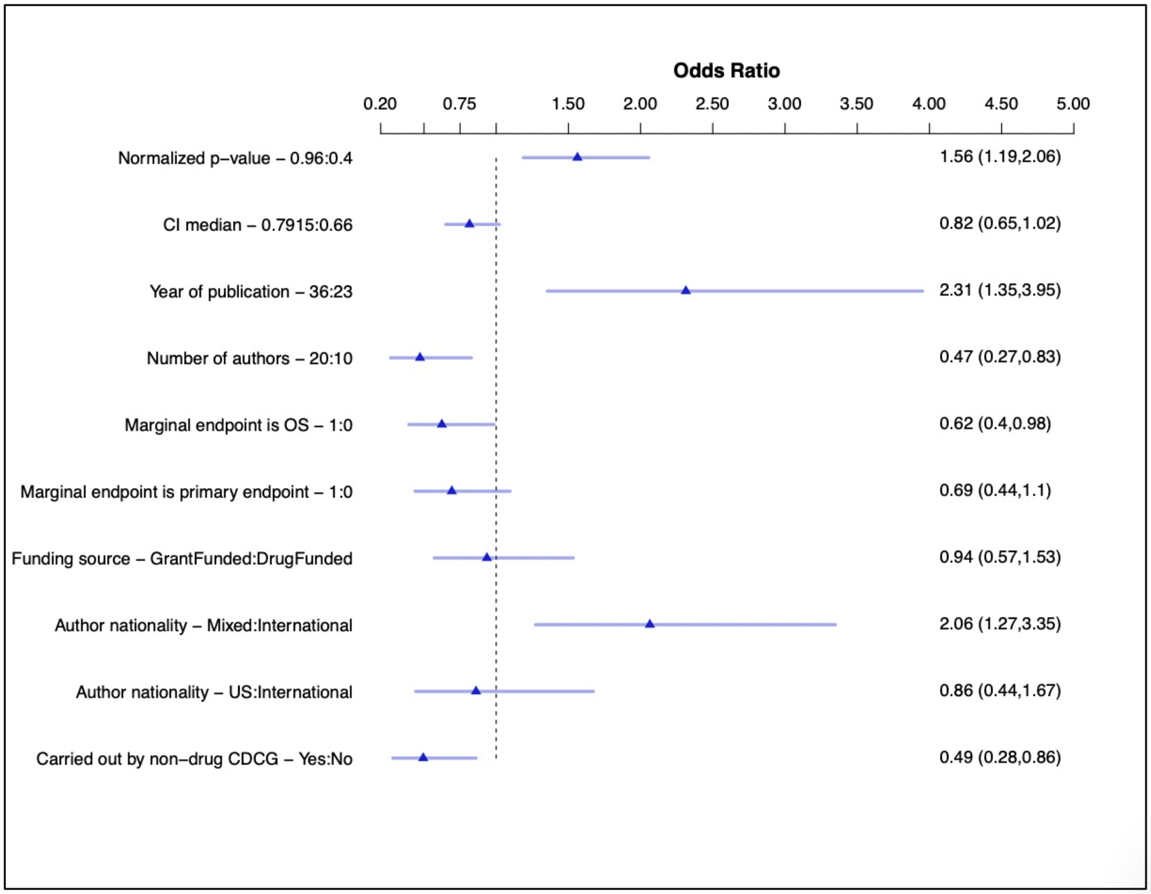

Forest plot demonstrating the magnitude of effect of each categorical covariate on uncertainty expression in the subgroup analysis. Shaded error bars indicate the 95% CI's around each odds ratio graphically.

eTable 1. Example[a] labeling of comparative efficacy of superiority trials on HemOnc.org.

**Primary outcome of PFS is…**	**…and secondary outcome of OS[b] is…**	**…then the label on *HemOnc.org* is:[c]**
Negative (P = 0.33)	Negative (any P)	Did not meet primary endpoint of PFS
Negative (P = 0.33)	Positive (any P)	Did not meet primary endpoint of PFS
Negative (P = 0.09)	Negative (any P)	Might have superior PFS
Negative (P = 0.09)	Positive (any P)	Might have superior PFS
Positive (P = 0.03)	Negative (P = 0.33)	Seems to have superior PFS
Positive (P = 0.03)	Negative (P = 0.09)	Might have superior OS
Positive (P = 0.03)	Positive (P = 0.03)	Seems to have superior OS
Positive (P = 0.03)	Positive (P = 0.0001)	Superior OS
Positive (P = 0.0001)	Negative (P = 0.33)	Superior PFS
Positive (P = 0.0001)	Negative (P = 0.09)	Might have superior OS
Positive (P = 0.0001)	Positive (P = 0.03)	Seems to have superior OS
Positive (P = 0.0001)	Positive (P = 0.0001)	Superior OS

[a]In these examples, the primary outcome is progression-free survival (PFS) and secondary outcome is overall survival (OS).
[b]Note that because OS is a secondary outcome, it is not possible to have a label of "might have superior OS".
[c]Positive outcomes are labeled from the perspective of the superior arm; the language for the inferior arm is identical with word "inferior" substituted.

© 2019 Rubinstein SM et al. *JAMA Network Open.*

eTable 2. Inclusion of marginal initial and subsequent analyses.

**Initial abstract statistical significance**	**Subsequent abstract statistical significance**	**Abstract included in this analysis**
0.01 – 0.05	<0.01	**Initial abstract**
<0.01	0.01 – 0.05	**Subsequent abstract**
0.01-0.05	0.01-0.05	**Initial abstract**

The initial publication of a trial is the first publication in a peer-reviewed journal (i.e., conference abstracts are excluded).

© 2019 Rubinstein SM et al. *JAMA Network Open.*

eTable 3. Journal Tiers as defined in this analysis

**Tier**	**Journals**
Tier 1: high-impact general science or medical journals	*Nature*<br>*Science*<br>*The Lancet*<br>*The New England Journal of Medicine*<br>*JAMA: the Journal of the American Medical Association*
Tier 2: high-impact specialty-specific medical journals	*Journal of Clinical Oncology*<br>*Blood*<br>*Lancet Oncology*<br>*Annals of Oncology*<br>*JAMA Oncology*<br>*Lancet Haematology*<br>*Annals of Internal Medicine*<br>*Annals of Neurology*<br>*Clinical Cancer Research*<br>*Journal of the National Cancer Institute*<br>*Leukemia*<br>*Journal of Thoracic Oncology*
Tier 3: lower impact specialty-specific medical journals	All other journals

© 2019 Rubinstein SM et al. *JAMA Network Open.*

eTable 4. Scoring Algorithm for confidence intervals

Score	Description
Two	The article expresses a confidence interval around a measure of comparative efficacy (such as a hazard ratio for overall survival, or odds ratio of a pathologic complete response)
One	The article reports confidence intervals for the results of individual arms but not for a measure of comparative efficacy
Zero	The article does not express confidence intervals around the marginal result in any capacity

© 2019 Rubinstein SM et al. *JAMA Network Open.*

eTable 5. Efficacy labels for trials on HemOnc.org

	P < 0.01	P 0.01 – 0.05 “Seems to have”	P 0.05-0.10 “Might have”	P > 0.10 “Did not meet primary endpoint”
**Superior**	938	652	172	2437
**Inferior**	709	557	192	

Comment: The imbalance between superior and inferior labels is due to trials having multiple arms, non-inclusion on the website, or both. For example, if the control arm of an RCT is superior, the inferior experimental arm(s) will not be included on the website, leading to a relative imbalance of superior labels.

© 2019 Rubinstein SM et al. *JAMA Network Open.*

eTable 6. PMIDs and titles of examined abstracts

PMID	Title
110744	Hydroxyurea or placebo combined with radiation to treat stages IIIB and IV cervical cancer confined to the pelvis.
194679	L-phenylalanine mustard (L-PAM) in the management of primary breast cancer. An update of earlier findings and a comparison with those utilizing L-PAM plus 5-fluorouracil (5-FU).
28843768	Carfilzomib or bortezomib in relapsed or refractory multiple myeloma (ENDEAVOR): an interim overall survival analysis of an open-label, randomised, phase 3 trial.
29175149	Neoadjuvant trastuzumab, pertuzumab, and chemotherapy versus trastuzumab emtansine plus pertuzumab in patients with HER2-positive breast cancer (KRISTINE): a randomised, open-label, multicentre, phase 3 trial.
21724462	Bortezomib plus rituximab versus rituximab alone in patients with relapsed, rituximab-naive or rituximab-sensitive, follicular lymphoma: a randomised phase 3 trial.
31175001	Quizartinib versus salvage chemotherapy in relapsed or refractory FLT3-ITD acute myeloid leukaemia (QuANTUM-R): a multicentre, randomised, controlled, open-label, phase 3 trial.
30922733	Capecitabine compared with observation in resected biliary tract cancer (BILCAP): a randomised, controlled, multicentre, phase 3 study.
28734822	Icotinib versus whole-brain irradiation in patients with EGFR-mutant non-small-cell lung cancer and multiple brain metastases (BRAIN): a multicentre, phase 3, open-label, parallel, randomised controlled trial.
27083332	Ponatinib versus imatinib for newly diagnosed chronic myeloid leukaemia: an international, randomised, open-label, phase 3 trial.
28017406	Bortezomib with lenalidomide and dexamethasone versus lenalidomide and dexamethasone alone in patients with newly diagnosed myeloma without intent for immediate autologous stem-cell transplant (SWOG S0777): a randomised, open-label, phase 3 trial.
31122901	Atezolizumab in combination with carboplatin plus nab-paclitaxel chemotherapy compared with chemotherapy alone as first-line treatment for metastatic non-squamous non-small-cell lung cancer (IMpower130): a multicentre, randomised, open-label, phase 3 trial.
24717919	Systemic chemotherapy with or without cetuximab in patients with resectable colorectal liver metastasis: the New EPOC randomised controlled trial.
27324280	Zoledronate in combination with chemotherapy and surgery to treat osteosarcoma (OS2006): a randomised, multicentre, open-label, phase 3 trial.
28438473	Bevacizumab and paclitaxel-carboplatin chemotherapy and secondary cytoreduction in recurrent, platinum-sensitive ovarian cancer (NRG Oncology/Gynecologic Oncology Group study GOG-0213): a multicentre, open-label, randomised, phase 3 trial.
30975627	Erlotinib plus bevacizumab versus erlotinib alone in patients with EGFR-positive advanced non-squamous non-small-cell lung cancer (NEJ026): interim analysis of an open-label, randomised, multicentre, phase 3 trial.
26045340	Necitumumab plus gemcitabine and cisplatin versus gemcitabine and cisplatin alone as first-line therapy in patients with stage IV squamous non-small-cell lung cancer (SQUIRE): an open-label, randomised, controlled phase 3 trial.
30665869	Ramucirumab after sorafenib in patients with advanced hepatocellular carcinoma and increased Œ±-fetoprotein concentrations (REACH-2): a randomised, double-blind, placebo-controlled, phase 3 trial.
31036468	Tucidinostat plus exemestane for postmenopausal patients with advanced, hormone receptor-positive breast cancer (ACE): a randomised, double-blind, placebo-controlled, phase 3 trial.

© 2019 Rubinstein SM et al. *JAMA Network Open.*

28882536	Gemcitabine and docetaxel versus doxorubicin as first-line treatment in previously untreated advanced unresectable or metastatic soft-tissue sarcomas (GeDDiS): a randomised controlled phase 3 trial.
29103871	Olaparib in combination with paclitaxel in patients with advanced gastric cancer who have progressed following first-line therapy (GOLD): a double-blind, randomised, placebo-controlled, phase 3 trial.
20434400	Neo-adjuvant chemotherapy alone or with regional hyperthermia for localised high-risk soft-tissue sarcoma: a randomised phase 3 multicentre study.
26590673	Standard first-line chemotherapy with or without nintedanib for advanced ovarian cancer (AGO-OVAR 12): a randomised, double-blind, placebo-controlled phase 3 trial.
27374467	The effect of salvage autologous stem-cell transplantation on overall survival in patients with relapsed multiple myeloma (final results from BSBMT/UKMF Myeloma X Relapse [Intensive]): a randomised, open-label, phase 3 trial.
26189067	Oxaliplatin added to fluorouracil-based preoperative chemoradiotherapy and postoperative chemotherapy of locally advanced rectal cancer (the German CAO/ARO/AIO-04 study): final results of the multicentre, open-label, randomised, phase 3 trial.
30217672	Pertuzumab plus trastuzumab and chemotherapy for HER2-positive metastatic gastric or gastro-oesophageal junction cancer (JACOB): final analysis of a double-blind, randomised, placebo-controlled phase 3 study.
27155740	Timing of androgen-deprivation therapy in patients with prostate cancer with a rising PSA (TROG 03.06 and VCOG PR 01-03 [TOAD]): a randomised, multicentre, non-blinded, phase 3 trial.
22627104	Preoperative chemoradiotherapy and postoperative chemotherapy with fluorouracil and oxaliplatin versus fluorouracil alone in locally advanced rectal cancer: initial results of the German CAO/ARO/AIO-04 randomised phase 3 trial.
11920457	Gemcitabine alone or with cisplatin for the treatment of patients with locally advanced and/or metastatic pancreatic carcinoma: a prospective, randomized phase III study of the Gruppo Oncologia dell'Italia Meridionale.
21131037	First-line treatment with zoledronic acid as compared with clodronic acid in multiple myeloma (MRC Myeloma IX): a randomised controlled trial.
27720136	IMA901, a multipeptide cancer vaccine, plus sunitinib versus sunitinib alone, as first-line therapy for advanced or metastatic renal cell carcinoma (IMPRINT): a multicentre, open-label, randomised, controlled, phase 3 trial.
26234174	Vosaroxin plus cytarabine versus placebo plus cytarabine in patients with first relapsed or refractory acute myeloid leukaemia (VALOR): a randomised, controlled, double-blind, multinational, phase 3 study.
24618336	Doxorubicin alone versus intensified doxorubicin plus ifosfamide for first-line treatment of advanced or metastatic soft-tissue sarcoma: a randomised controlled phase 3 trial.
25892145	Afatinib versus methotrexate as second-line treatment in patients with recurrent or metastatic squamous-cell carcinoma of the head and neck progressing on or after platinum-based therapy (LUX-Head & Neck 1): an open-label, randomised phase 3 trial.
29423520	Cisplatin Chemoradiotherapy vs Radiotherapy in FIGO Stage IIIB Squamous Cell Carcinoma of the Uterine Cervix: A Randomized Clinical Trial.
21377930	Zalutumumab plus best supportive care versus best supportive care alone in patients with recurrent or metastatic squamous-cell carcinoma of the head and neck after failure of platinum-based chemotherapy: an open-label, randomised phase 3 trial.
28916371	Ramucirumab plus docetaxel versus placebo plus docetaxel in patients with locally advanced or metastatic urothelial carcinoma after platinum-based therapy (RANGE): a randomised, double-blind, phase 3 trial.

© 2019 Rubinstein SM et al. *JAMA Network Open.*

23615461	Rituximab plus cyclophosphamide, doxorubicin, vincristine, and prednisolone in patients with newly diagnosed diffuse large B-cell non-Hodgkin lymphoma: a phase 3 comparison of dose intensification with 14-day versus 21-day cycles.
26429297	Busulfan plus cyclophosphamide versus busulfan plus fludarabine as a preparative regimen for allogeneic haemopoietic stem-cell transplantation in patients with acute myeloid leukaemia: an open-label, multicentre, randomised, phase 3 trial.
26474518	Bevacizumab with or without erlotinib as maintenance therapy in patients with metastatic colorectal cancer (GERCOR DREAM; OPTIMOX3): a randomised, open-label, phase 3 trial.
27080498	Rituximab and dose-dense chemotherapy for adults with Burkitt's lymphoma: a randomised, controlled, open-label, phase 3 trial.
22192731	Gemcitabine and oxaliplatin with or without erlotinib in advanced biliary-tract cancer: a multicentre, open-label, randomised, phase 3 study.
27686945	Induction chemotherapy plus concurrent chemoradiotherapy versus concurrent chemoradiotherapy alone in locoregionally advanced nasopharyngeal carcinoma: a phase 3, multicentre, randomised controlled trial.
8656680	Prednimustine, mitoxantrone (PmM) vs cyclophosphamide, vincristine, prednisone (COP) for the treatment of advanced low-grade non-Hodgkin's lymphoma. German Low-Grade Lymphoma Study Group.
21233014	Induction chemotherapy with cisplatin and fluorouracil alone or in combination with docetaxel in locally advanced squamous-cell cancer of the head and neck: long-term results of the TAX 324 randomised phase 3 trial.
31079938	Atezolizumab plus bevacizumab versus sunitinib in patients with previously untreated metastatic renal cell carcinoma (IMmotion151): a multicentre, open-label, phase 3, randomised controlled trial.
26522337	Nedaplatin plus docetaxel versus cisplatin plus docetaxel for advanced or relapsed squamous cell carcinoma of the lung (WJOG5208L): a randomised, open-label, phase 3 trial.
8901852	Randomized trial of two versus five years of adjuvant tamoxifen for postmenopausal early stage breast cancer. Swedish Breast Cancer Cooperative Group.
24206640	Axitinib versus sorafenib as first-line therapy in patients with metastatic renal-cell carcinoma: a randomised open-label phase 3 trial.
12377641	Phase III randomised trial comparing paclitaxel/carboplatin with paclitaxel/cisplatin in patients with advanced non-small-cell lung cancer: a cooperative multinational trial.
30509771	Use of letrozole after aromatase inhibitor-based therapy in postmenopausal breast cancer (NRG Oncology/NSABP B-42): a randomised, double-blind, placebo-controlled, phase 3 trial.
26338525	FOLFOXIRI plus bevacizumab versus FOLFIRI plus bevacizumab as first-line treatment of patients with metastatic colorectal cancer: updated overall survival and molecular subgroup analyses of the open-label, phase 3 TRIBE study.
23782814	Intercalated combination of chemotherapy and erlotinib for patients with advanced stage non-small-cell lung cancer (FASTACT-2): a randomised, double-blind trial.
26719230	Bevacizumab for newly diagnosed pleural mesothelioma in the Mesothelioma Avastin Cisplatin Pemetrexed Study (MAPS): a randomised, controlled, open-label, phase 3 trial.
28359784	Ipilimumab 10 mg/kg versus ipilimumab 3 mg/kg in patients with unresectable or metastatic melanoma: a randomised, double-blind, multicentre, phase 3 trial.
30910541	Conditioning with busulfan plus melphalan versus melphalan alone before autologous haemopoietic cell transplantation for multiple myeloma: an open-label, randomised, phase 3 trial.

© 2019 Rubinstein SM et al. *JAMA Network Open.*

30782343	Lomustine-temozolomide combination therapy versus standard temozolomide therapy in patients with newly diagnosed glioblastoma with methylated MGMT promoter (CeTeG/NOA-09): a randomised, open-label, phase 3 trial.
22622008	Thoracic radiotherapy with or without daily low-dose carboplatin in elderly patients with non-small-cell lung cancer: a randomised, controlled, phase 3 trial by the Japan Clinical Oncology Group (JCOG0301).
24831977	Ipilimumab versus placebo after radiotherapy in patients with metastatic castration-resistant prostate cancer that had progressed after docetaxel chemotherapy (CA184-043): a multicentre, randomised, double-blind, phase 3 trial.
27313086	Addition of high-dose cytarabine to immunochemotherapy before autologous stem-cell transplantation in patients aged 65 years or younger with mantle cell lymphoma (MCL Younger): a randomised, open-label, phase 3 trial of the European Mantle Cell Lymphoma Network.
17227978	Adjuvant chemotherapy with gemcitabine vs observation in patients undergoing curative-intent resection of pancreatic cancer: a randomized controlled trial.
25864104	Ombrabulin plus cisplatin versus placebo plus cisplatin in patients with advanced soft-tissue sarcomas after failure of anthracycline and ifosfamide chemotherapy: a randomised, double-blind, placebo-controlled, phase 3 trial.
24055414	Vorinostat or placebo in combination with bortezomib in patients with multiple myeloma (VANTAGE 088): a multicentre, randomised, double-blind study.
18282805	S-1 plus cisplatin versus S-1 alone for first-line treatment of advanced gastric cancer (SPIRITS trial): a phase III trial.
25240821	Ramucirumab plus paclitaxel versus placebo plus paclitaxel in patients with previously treated advanced gastric or gastro-oesophageal junction adenocarcinoma (RAINBOW): a double-blind, randomised phase 3 trial.
26028518	Androgen deprivation therapy plus docetaxel and estramustine versus androgen deprivation therapy alone for high-risk localised prostate cancer (GETUG 12): a phase 3 randomised controlled trial.
30522922	Brentuximab vedotin with chemotherapy for CD30-positive peripheral T-cell lymphoma (ECHELON-2): a global, double-blind, randomised, phase 3 trial.
3519883	Ten-year results from the National Surgical Adjuvant Breast and Bowel Project (NSABP) clinical trial evaluating the use of L-phenylalanine mustard (L-PAM) in the management of primary breast cancer.
8780630	Improved survival in stage III non-small-cell lung cancer: seven-year follow-up of cancer and leukemia group B (CALGB) 8433 trial.
19897418	Radiotherapy plus cetuximab for locoregionally advanced head and neck cancer: 5-year survival data from a phase 3 randomised trial, and relation between cetuximab-induced rash and survival.
26037941	Dabrafenib and trametinib versus dabrafenib and placebo for Val600 BRAF-mutant melanoma: a multicentre, double-blind, phase 3 randomised controlled trial.
19767092	Dose-dense paclitaxel once a week in combination with carboplatin every 3 weeks for advanced ovarian cancer: a phase 3, open-label, randomised controlled trial.
15520061	Randomised phase III study of intravenous vinorelbine plus hormone therapy versus hormone therapy alone in hormone-refractory prostate cancer.
18784101	Platinum-based chemotherapy plus cetuximab in head and neck cancer.
20631341	Comparison of two standard chemotherapy regimens for good-prognosis germ cell tumors: updated analysis of a randomized trial.
28129987	Comparison of adjuvant gemcitabine and capecitabine with gemcitabine monotherapy in patients with resected pancreatic cancer (ESPAC-4): a multicentre, open-label, randomised, phase 3 trial.

© 2019 Rubinstein SM et al. *JAMA Network Open.*

22652183	Pixantrone dimaleate versus other chemotherapeutic agents as a single-agent salvage treatment in patients with relapsed or refractory aggressive non-Hodgkin lymphoma: a phase 3, multicentre, open-label, randomised trial.
27908454	Fulvestrant 500 mg versus anastrozole 1 mg for hormone receptor-positive advanced breast cancer (FALCON): an international, randomised, double-blind, phase 3 trial.
24104372	Adjuvant chemotherapy with gemcitabine and long-term outcomes among patients with resected pancreatic cancer: the CONKO-001 randomized trial.
29450452	Effect of Neoadjuvant Chemotherapy Plus Regional Hyperthermia on Long-term Outcomes Among Patients With Localized High-Risk Soft Tissue Sarcoma: The EORTC 62961-ESHO 95 Randomized Clinical Trial.
22474202	Effect of oxaliplatin, fluorouracil, and leucovorin with or without cetuximab on survival among patients with resected stage III colon cancer: a randomized trial.
8774567	Comparison of oral etoposide and standard intravenous multidrug chemotherapy for small-cell lung cancer: a stopped multicentre randomised trial. Medical Research Council Lung Cancer Working Party.
21992852	Fludarabine plus alemtuzumab versus fludarabine alone in patients with previously treated chronic lymphocytic leukaemia: a randomised phase 3 trial.
23234763	Adjuvant lapatinib for women with early-stage HER2-positive breast cancer: a randomised, controlled, phase 3 trial.
24933332	Ramucirumab plus docetaxel versus placebo plus docetaxel for second-line treatment of stage IV non-small-cell lung cancer after disease progression on platinum-based therapy (REVEL): a multicentre, double-blind, randomised phase 3 trial.
24556040	Dovitinib versus sorafenib for third-line targeted treatment of patients with metastatic renal cell carcinoma: an open-label, randomised phase 3 trial.
30982686	Perioperative chemotherapy with fluorouracil plus leucovorin, oxaliplatin, and docetaxel versus fluorouracil or capecitabine plus cisplatin and epirubicin for locally advanced, resectable gastric or gastro-oesophageal junction adenocarcinoma (FLOT4): a randomised, phase 2/3 trial.
29880231	Pembrolizumab versus paclitaxel for previously treated, advanced gastric or gastro-oesophageal junction cancer (KEYNOTE-061): a randomised, open-label, controlled, phase 3 trial.
10679658	Mature results of a phase III randomized trial comparing concurrent chemoradiotherapy with radiation therapy alone in patients with stage III and IV squamous cell carcinoma of the head and neck.
19095497	Efficacy and safety of sorafenib in patients in the Asia-Pacific region with advanced hepatocellular carcinoma: a phase III randomised, double-blind, placebo-controlled trial.
26874885	Eribulin versus dacarbazine in previously treated patients with advanced liposarcoma or leiomyosarcoma: a randomised, open-label, multicentre, phase 3 trial.
11063636	A phase III trial of ifosfamide with or without cisplatin in carcinosarcoma of the uterus: A Gynecologic Oncology Group Study.
30188789	High-Dose Chemotherapy and Blood Autologous Stem-Cell Rescue Compared With Standard Chemotherapy in Localized High-Risk Ewing Sarcoma: Results of Euro-E.W.I.N.G.99 and Ewing-2008.
22204725	A phase 3 trial of bevacizumab in ovarian cancer.
23294853	2-Weekly versus 3-weekly docetaxel to treat castration-resistant advanced prostate cancer: a randomised, phase 3 trial.
18695132	Radical prostatectomy versus watchful waiting in localized prostate cancer: the Scandinavian prostate cancer group-4 randomized trial.
22056152	Combined androgen deprivation therapy and radiation therapy for locally advanced prostate cancer: a randomised, phase 3 trial.

© 2019 Rubinstein SM et al. *JAMA Network Open.*

22482940	Effect of gemtuzumab ozogamicin on survival of adult patients with de-novo acute myeloid leukaemia (ALFA-0701): a randomised, open-label, phase 3 study.
25975632	Efficacy of neoadjuvant bevacizumab added to docetaxel followed by fluorouracil, epirubicin, and cyclophosphamide, for women with HER2-negative early breast cancer (ARTemis): an open-label, randomised, phase 3 trial.
1702144	Superiority of ProMACE-CytaBOM over ProMACE-MOPP in the treatment of advanced diffuse aggressive lymphoma: results of a prospective randomized trial.
29224502	Brentuximab Vedotin with Chemotherapy for Stage III or IV Hodgkin's Lymphoma.
30917258	Overall Survival with Fulvestrant plus Anastrozole in Metastatic Breast Cancer.
15470214	Docetaxel and estramustine compared with mitoxantrone and prednisone for advanced refractory prostate cancer.
26324362	Capecitabine Plus Oxaliplatin Compared With Fluorouracil/Folinic Acid As Adjuvant Therapy for Stage III Colon Cancer: Final Results of the NO16968 Randomized Controlled Phase III Trial.
24297940	High-dose cytarabine in induction treatment improves the outcome of adult patients younger than age 46 years with acute myeloid leukemia: results of the EORTC-GIMEMA AML-12 trial.
7509381	A randomized study in stage IIIB and IV Hodgkin's disease comparing eight courses of MOPP versus an alteration of MOPP with ABVD: a European Organization for Research and Treatment of Cancer Lymphoma Cooperative Group and Groupe Pierre-et-Marie-Curie controlled clinical trial.
27292104	Inotuzumab Ozogamicin versus Standard Therapy for Acute Lymphoblastic Leukemia.
29031778	Extended adjuvant aromatase inhibition after sequential endocrine therapy (DATA): a randomised, phase 3 trial.
2189950	Postoperative chemotherapy and tamoxifen compared with tamoxifen alone in the treatment of positive-node breast cancer patients aged 50 years and older with tumors responsive to tamoxifen: results from the National Surgical Adjuvant Breast and Bowel Project B-16.
12529344	Impact of adjuvant chemotherapy and surgical staging in early-stage ovarian carcinoma: European Organisation for Research and Treatment of Cancer-Adjuvant ChemoTherapy in Ovarian Neoplasm trial.
9508168	Mitoxantrone versus daunorubicin in induction-consolidation chemotherapy--the value of low-dose cytarabine for maintenance of remission, and an assessment of prognostic factors in acute myeloid leukemia in the elderly: final report. European Organization for the Research and Treatment of Cancer and the Dutch-Belgian Hemato-Oncology Cooperative Hovon Group.
29220295	Once-a-Week Versus Once-Every-3-Weeks Cisplatin Chemoradiation for Locally Advanced Head and Neck Cancer: A Phase III Randomized Noninferiority Trial.
8676625	Multicentre prospective randomised trial of fludarabine versus cyclophosphamide, doxorubicin, and prednisone (CAP) for treatment of advanced-stage chronic lymphocytic leukaemia. The French Cooperative Group on CLL.
29241450	Azacitidine improves clinical outcomes in older patients with acute myeloid leukaemia with myelodysplasia-related changes compared with conventional care regimens.
23088650	Bortezomib plus rituximab versus rituximab in patients with high-risk, relapsed, rituximab-na√Øve or rituximab-sensitive follicular lymphoma: subgroup analysis of a randomized phase 3 trial.
28892431	Nintedanib Plus Pemetrexed/Cisplatin in Patients With Malignant Pleural Mesothelioma: Phase II Results From the Randomized, Placebo-Controlled LUME-Meso Trial.

© 2019 Rubinstein SM et al. *JAMA Network Open.*

8616766	Randomized trial of initial therapy with melphalan versus cisplatin-based combination chemotherapy in patients with advanced ovarian carcinoma: initial and long term results--Eastern Cooperative Oncology Group Study E2878.
24094768	Ramucirumab monotherapy for previously treated advanced gastric or gastro-oesophageal junction adenocarcinoma (REGARD): an international, randomised, multicentre, placebo-controlled, phase 3 trial.
25877855	Ramucirumab versus placebo in combination with second-line FOLFIRI in patients with metastatic colorectal carcinoma that progressed during or after first-line therapy with bevacizumab, oxaliplatin, and a fluoropyrimidine (RAISE): a randomised, double-blind, multicentre, phase 3 study.
28437161	Randomized Phase III Trial of Trastuzumab Plus Capecitabine With or Without Pertuzumab in Patients With Human Epidermal Growth Factor Receptor 2-Positive Metastatic Breast Cancer Who Experienced Disease Progression During or After Trastuzumab-Based Therapy.
20942865	Addition of thalidomide to oral melphalan/prednisone in patients with multiple myeloma not eligible for transplantation: results of a randomized trial from the Turkish Myeloma Study Group.
15128893	Postoperative concurrent radiotherapy and chemotherapy for high-risk squamous-cell carcinoma of the head and neck.
29215955	Results of a Randomized, Double-Blind, Placebo-Controlled, Phase III Trial of Trifluridine/Tipiracil (TAS-102) Monotherapy in Asian Patients With Previously Treated Metastatic Colorectal Cancer: The TERRA Study.
29878867	Improved Outcome by Adding Concurrent Chemotherapy to Cetuximab and Radiotherapy for Locally Advanced Head and Neck Carcinomas: Results of the GORTEC 2007-01 Phase III Randomized Trial.
21376385	Eribulin monotherapy versus treatment of physician's choice in patients with metastatic breast cancer (EMBRACE): a phase 3 open-label randomised study.
27138575	Phase III Trial Evaluating Letrozole As First-Line Endocrine Therapy With or Without Bevacizumab for the Treatment of Postmenopausal Women With Hormone Receptor-Positive Advanced-Stage Breast Cancer: CALGB 40503 (Alliance).
29091516	Bosutinib Versus Imatinib for Newly Diagnosed Chronic Myeloid Leukemia: Results From the Randomized BFORE Trial.
9827708	OK-432 and 5-fluorouracil, doxorubicin, and mitomycin C (FAM-P) versus FAM chemotherapy in patients with curatively resected gastric carcinoma: a randomized Phase III trial.
30089078	Neoadjuvant Chemoradiotherapy Followed by Surgery Versus Surgery Alone for Locally Advanced Squamous Cell Carcinoma of the Esophagus (NEOCRTEC5010): A Phase III Multicenter, Randomized, Open-Label Clinical Trial.
10811675	Prospective randomized trial of docetaxel versus best supportive care in patients with non-small-cell lung cancer previously treated with platinum-based chemotherapy.
28953447	Rituximab after Autologous Stem-Cell Transplantation in Mantle-Cell Lymphoma.
30920645	Inotuzumab ozogamicin versus standard of care in relapsed or refractory acute lymphoblastic leukemia: Final report and long-term survival follow-up from the randomized, phase 3 INO-VATE study.
30241001	Buparlisib plus fulvestrant versus placebo plus fulvestrant for postmenopausal, hormone receptor-positive, human epidermal growth factor receptor 2-negative, advanced breast cancer: Overall survival results from BELLE-2.
20888994	Addition of rituximab to fludarabine and cyclophosphamide in patients with chronic lymphocytic leukaemia: a randomised, open-label, phase 3 trial.
28581356	Adjuvant Pertuzumab and Trastuzumab in Early HER2-Positive Breast Cancer.

9389919	A dose-controlled study of 153Sm-ethylenediaminetetramethylenephosphonate (EDTMP) in the treatment of patients with painful bone metastases.
21149672	Immunosuppressive therapy for patients with myelodysplastic syndrome: a prospective randomized multicenter phase III trial comparing antithymocyte globulin plus cyclosporine with best supportive care--SAKK 33/99.
22873532	Treatment of older patients with mantle-cell lymphoma.
1730080	Cytarabine plus idarubicin or daunorubicin as induction and consolidation therapy for previously untreated adult patients with acute myeloid leukemia.
1635566	Treatment of metastatic malignant melanoma with dacarbazine plus tamoxifen.
8080680	Neoadjuvant versus adjuvant chemotherapy in premenopausal patients with tumours considered too large for breast conserving surgery: preliminary results of a randomised trial: S6.
15128894	Postoperative irradiation with or without concomitant chemotherapy for locally advanced head and neck cancer.
15888698	Radical prostatectomy versus watchful waiting in early prostate cancer.
18620949	Adjuvant therapy with pegylated interferon alfa-2b versus observation alone in resected stage III melanoma: final results of EORTC 18991, a randomised phase III trial.
27056996	A randomized phase III trial comparing S-1 versus UFT as adjuvant chemotherapy for stage II/III rectal cancer (JFMC35-C1: ACTS-RC).
29584548	Overall Survival Benefit in Patients With Rituximab-Refractory Indolent Non-Hodgkin Lymphoma Who Received Obinutuzumab Plus Bendamustine Induction and Obinutuzumab Maintenance in the GADOLIN Study.
9323144	A phase III randomized trial comparing concurrent chemotherapy and radiotherapy with radiotherapy alone in resectable stage III and IV squamous cell head and neck cancer: preliminary results.
23150707	Long-term update of US GI intergroup RTOG 98-11 phase III trial for anal carcinoma: survival, relapse, and colostomy failure with concurrent chemoradiation involving fluorouracil/mitomycin versus fluorouracil/cisplatin.
23643177	Randomized phase III trial of gemcitabine and cisplatin vs. gemcitabine alone in patients with advanced non-small cell lung cancer and a performance status of 2: the CAPPA-2 study.
19767093	Maintenance pemetrexed plus best supportive care versus placebo plus best supportive care for non-small-cell lung cancer: a randomised, double-blind, phase 3 study.
22149921	ABVD alone versus radiation-based therapy in limited-stage Hodgkin's lymphoma.
22453568	One vs three years of adjuvant imatinib for operable gastrointestinal stromal tumor: a randomized trial.
29746229	Phase III Trial Comparing Intraperitoneal and Intravenous Paclitaxel Plus S-1 Versus Cisplatin Plus S-1 in Patients With Gastric Cancer With Peritoneal Metastasis: PHOENIX-GC Trial.
26861603	Observation versus late reintroduction of letrozole as adjuvant endocrine therapy for hormone receptor-positive breast cancer (ANZ0501 LATER): an open-label randomised, controlled trial.
29395502	Docetaxel Versus Surveillance After Radical Prostatectomy for High-risk Prostate Cancer: Results from the Prospective Randomised, Open-label Phase 3 Scandinavian Prostate Cancer Group 12 Trial.
26527776	Adjuvant Fluorouracil, Leucovorin, and Oxaliplatin in Stage II to III Colon Cancer: Updated 10-Year Survival and Outcomes According to BRAF Mutation and Mismatch Repair Status of the MOSAIC Study.
25600568	Continuation or reintroduction of bevacizumab beyond progression to first-line therapy in metastatic colorectal cancer: final results of the randomized BEBYP trial.

16198768	Post-surgery adjuvant therapy with intermediate doses of interferon alfa 2b versus observation in patients with stage IIb/III melanoma (EORTC 18952): randomised controlled trial.
30138085	Improved Survival for Children and Young Adults With T-Lineage Acute Lymphoblastic Leukemia: Results From the Children's Oncology Group AALL0434 Methotrexate Randomization.
23948349	Long-term results of dose-dense paclitaxel and carboplatin versus conventional paclitaxel and carboplatin for treatment of advanced epithelial ovarian, fallopian tube, or primary peritoneal cancer (JGOG 3016): a randomised, controlled, open-label trial.
2458438	A randomized trial of two dose levels of cyclophosphamide, methotrexate, and fluorouracil chemotherapy for patients with metastatic breast cancer.
17960012	Cisplatin, fluorouracil, and docetaxel in unresectable head and neck cancer.
28146658	Radiation with or without Antiandrogen Therapy in Recurrent Prostate Cancer.
19687335	Phase III trial of vinflunine plus best supportive care compared with best supportive care alone after a platinum-containing regimen in patients with advanced transitional cell carcinoma of the urothelial tract.
26216386	Comparison of two different S-1 plus cisplatin dosing schedules as first-line chemotherapy for metastatic and/or recurrent gastric cancer: a multicenter, randomized phase III trial (SOS).
29864379	Improvement in Overall Survival in a Randomized Study That Compared Dacomitinib With Gefitinib in Patients With Advanced Non-Small-Cell Lung Cancer and EGFR-Activating Mutations.
24145346	PointBreak: a randomized phase III study of pemetrexed plus carboplatin and bevacizumab followed by maintenance pemetrexed and bevacizumab versus paclitaxel plus carboplatin and bevacizumab followed by maintenance bevacizumab in patients with stage IIIB or IV nonsquamous non-small-cell lung cancer.
18779613	Irinotecan plus carboplatin versus oral etoposide plus carboplatin in extensive small-cell lung cancer: a randomized phase III trial.
10615075	Hepatic arterial infusion of chemotherapy after resection of hepatic metastases from colorectal cancer.
24827123	ABVD (8 cycles) versus BEACOPP (4 escalated cycles ,â• 4 baseline): final results in stage III-IV low-risk Hodgkin lymphoma (IPS 0-2) of the LYSA H34 randomized trial.
18032763	Lenalidomide plus dexamethasone for relapsed multiple myeloma in North America.
16394300	Intraperitoneal cisplatin and paclitaxel in ovarian cancer.
28911070	Induction TPF followed by concomitant treatment versus concomitant treatment alone in locally advanced head and neck cancer. A phase II-III trial.
28902534	Phase III, Randomized, Placebo-Controlled, Double-Blind Trial of Motesanib (AMG-706) in Combination With Paclitaxel and Carboplatin in East Asian Patients With Advanced Nonsquamous Non-Small-Cell Lung Cancer.
28463633	Dose-Reduced Versus Standard Conditioning Followed by Allogeneic Stem-Cell Transplantation for Patients With Myelodysplastic Syndrome: A Prospective Randomized Phase III Study of the EBMT (RICMAC Trial).
28426350	Lenalidomide Maintenance Compared With Placebo in Responding Elderly Patients With Diffuse Large B-Cell Lymphoma Treated With First-Line Rituximab Plus Cyclophosphamide, Doxorubicin, Vincristine, and Prednisone.
22434360	Pemetrexed in combination with cisplatin versus cisplatin monotherapy in patients with recurrent or metastatic head and neck cancer: final results of a randomized, double-blind, placebo-controlled, phase 3 study.
12902441	Randomized phase III trial of paclitaxel, etoposide, and carboplatin versus carboplatin, etoposide, and vincristine in patients with small-cell lung cancer.

23233721	Results of a randomized trial of chlorambucil versus fludarabine for patients with untreated Waldenstr√∂m macroglobulinemia, marginal zone lymphoma, or lymphoplasmacytic lymphoma.
25332243	Southwest Oncology Group S0008: a phase III trial of high-dose interferon Alfa-2b versus cisplatin, vinblastine, and dacarbazine, plus interleukin-2 and interferon in patients with high-risk melanoma--an intergroup study of cancer and leukemia Group B, Children's Oncology Group, Eastern Cooperative Oncology Group, and Southwest Oncology Group.
28228106	Long-term treatment with ruxolitinib for patients with myelofibrosis: 5-year update from the randomized, double-blind, placebo-controlled, phase 3 COMFORT-I trial.
23322524	Sequential docetaxel as adjuvant chemotherapy for node-positive or/and T3 or T4 breast cancer: clinical outcome (Mansoura University).
6336981	Chemotherapy versus combination of chemotherapy and endocrine therapy in advanced breast cancer. A prospective randomized study.
19439741	Adjuvant chemotherapy in older women with early-stage breast cancer.
29447329	Randomised phase III trial of vinflunine plus capecitabine versus capecitabine alone in patients with advanced breast cancer previously treated with an anthracycline and resistant to taxane.
3513075	Controlled phase III clinical study of 4-epi-doxorubicin + 5-fluorouracil versus 5-fluorouracil alone in metastatic gastric and rectosigmoid cancer.
11248153	Use of chemotherapy plus a monoclonal antibody against HER2 for metastatic breast cancer that overexpresses HER2.
28214653	Neoadjuvant chemotherapy followed by concurrent chemoradiotherapy versus concurrent chemoradiotherapy alone in locoregionally advanced nasopharyngeal carcinoma: A phase III multicentre randomised controlled trial.
22252613	A randomized study comparing yttrium-90 ibritumomab tiuxetan (Zevalin) and high-dose BEAM chemotherapy versus BEAM alone as the conditioning regimen before autologous stem cell transplantation in patients with aggressive lymphoma.
26014294	BEYOND: A Randomized, Double-Blind, Placebo-Controlled, Multicenter, Phase III Study of First-Line Carboplatin/Paclitaxel Plus Bevacizumab or Placebo in Chinese Patients With Advanced or Recurrent Nonsquamous Non-Small-Cell Lung Cancer.
22138037	A randomized phase III trial of combined paclitaxel, carboplatin, and radiation therapy followed by weekly paclitaxel or observation for patients with locally advanced inoperable non-small-cell lung cancer.
25349301	Phase III study of iniparib plus gemcitabine and carboplatin versus gemcitabine and carboplatin in patients with metastatic triple-negative breast cancer.
1922207	A randomized trial of intravesical doxorubicin and immunotherapy with bacille Calmette-Gu√©rin for transitional-cell carcinoma of the bladder.
27937096	Results From the Phase III Randomized Trial of Onartuzumab Plus Erlotinib Versus Erlotinib in Previously Treated Stage IIIB or IV Non-Small-Cell Lung Cancer: METLung.
2651576	Doxorubicin-containing regimens for the treatment of stage II breast cancer: The National Surgical Adjuvant Breast and Bowel Project experience.
26790144	Long term follow up of the EORTC 18952 trial of adjuvant therapy in resected stage IIB-III cutaneous melanoma patients comparing intermediate doses of interferon-alpha-2b (IFN) with observation: Ulceration of primary is key determinant for IFN-sensitivity.
23071247	Phase III trial of chemoradiotherapy for anaplastic oligodendroglioma: long-term results of RTOG 9402.
21444871	Phase III, open-label, randomized study comparing concurrent gemcitabine plus cisplatin and radiation followed by adjuvant gemcitabine and cisplatin versus

© 2019 Rubinstein SM et al. *JAMA Network Open.*

	concurrent cisplatin and radiation in patients with stage IIB to IVA carcinoma of the cervix.
29356608	Continued Excellent Outcomes in Previously Untreated Patients With Follicular Lymphoma After Treatment With CHOP Plus Rituximab or CHOP Plus ¹³¹I-Tositumomab: Long-Term Follow-Up of Phase III Randomized Study SWOG-S0016.
31171039	Final analyses of OPTiM: a randomized phase III trial of talimogene laherparepvec versus granulocyte-macrophage colony-stimulating factor in unresectable stage III-IV melanoma.
14517188	High-dose therapy improves progression-free survival and survival in relapsed follicular non-Hodgkin's lymphoma: results from the randomized European CUP trial.
18032762	Lenalidomide plus dexamethasone for relapsed or refractory multiple myeloma.
15352982	A randomized study (WOS MM1) comparing the oral regime Z-Dex (idarubicin and dexamethasone) with vincristine, adriamycin and dexamethasone as induction therapy for newly diagnosed patients with multiple myeloma.
22853014	Combination anastrozole and fulvestrant in metastatic breast cancer.
22802322	Bortezomib induction and maintenance treatment in patients with newly diagnosed multiple myeloma: results of the randomized phase III HOVON-65/ GMMG-HD4 trial.
23509322	Randomized trial of lapatinib versus placebo added to paclitaxel in the treatment of human epidermal growth factor receptor 2-overexpressing metastatic breast cancer.
2184356	Maintenance treatment with recombinant interferon alfa-2b in patients with multiple myeloma responding to conventional induction chemotherapy.
7909866	Randomised trial of monoclonal antibody for adjuvant therapy of resected Dukes' C colorectal carcinoma. German Cancer Aid 17-1A Study Group.
26527782	Adjuvant Imatinib for High-Risk GI Stromal Tumor: Analysis of a Randomized Trial.
28199818	Cabozantinib Versus Sunitinib As Initial Targeted Therapy for Patients With Metastatic Renal Cell Carcinoma of Poor or Intermediate Risk: The Alliance A031203 CABOSUN Trial.
15972865	Vinorelbine plus cisplatin vs. observation in resected non-small-cell lung cancer.
28662313	A multicenter, phase 3, randomized trial of concurrent chemoradiotherapy plus adjuvant chemotherapy versus radiotherapy alone in patients with regionally advanced nasopharyngeal carcinoma: 10-year outcomes for efficacy and toxicity.
11455971	A comparison of early intensive methotrexate/mercaptopurine with early intensive alternating combination chemotherapy for high-risk B-precursor acute lymphoblastic leukemia: a Pediatric Oncology Group phase III randomized trial.
9390536	Tamoxifen and chemotherapy for lymph node-negative, estrogen receptor-positive breast cancer.
10601378	Randomized trial of radiation therapy versus concomitant chemotherapy and radiation therapy for advanced-stage oropharynx carcinoma.
11138459	Epirubicin or epirubicin and cisplatin as first-line therapy in advanced breast cancer. A phase III study.
22851554	Addition of gemtuzumab ozogamicin to induction chemotherapy improves survival in older patients with acute myeloid leukemia.
24386911	Induction therapy with bortezomib and dexamethasone followed by autologous stem cell transplantation versus autologous stem cell transplantation alone in the treatment of renal AL amyloidosis: a randomized controlled trial.
25185099	Primary results of ROSE/TRIO-12, a randomized placebo-controlled phase III trial evaluating the addition of ramucirumab to first-line docetaxel chemotherapy in metastatic breast cancer.

© 2019 Rubinstein SM et al. *JAMA Network Open.*

28137739	Etoposide and cisplatin versus paclitaxel and carboplatin with concurrent thoracic radiotherapy in unresectable stage III non-small cell lung cancer: a multicenter randomized phase III trial.
2202789	Adjuvant therapy of Dukes' A, B, and C adenocarcinoma of the colon with portal-vein fluorouracil hepatic infusion: preliminary results of National Surgical Adjuvant Breast and Bowel Project Protocol C-02.
22512481	Radiotherapy with or without chemotherapy in muscle-invasive bladder cancer.
12840087	High-dose chemotherapy with hematopoietic stem-cell rescue for high-risk breast cancer.
19786658	Lapatinib combined with letrozole versus letrozole and placebo as first-line therapy for postmenopausal hormone receptor-positive metastatic breast cancer.
19269519	Preoperative radiotherapy versus selective postoperative chemoradiotherapy in patients with rectal cancer (MRC CR07 and NCIC-CTG C016): a multicentre, randomised trial.
23569301	Randomized controlled trial of cetuximab plus chemotherapy for patients with KRAS wild-type unresectable colorectal liver-limited metastases.
20567019	Phase III trial comparing protracted intravenous fluorouracil infusion alone or with yttrium-90 resin microspheres radioembolization for liver-limited metastatic colorectal cancer refractory to standard chemotherapy.
18794541	Adjuvant chemotherapy after potentially curative resection of metastases from colorectal cancer: a pooled analysis of two randomized trials.
29420221	Adjuvant Transarterial Chemoembolization for HBV-Related Hepatocellular Carcinoma After Resection: A Randomized Controlled Study.
12796019	Doxorubicin in combination with fluorouracil and cyclophosphamide (i.v. FAC regimen, day 1, 21) versus methotrexate in combination with fluorouracil and cyclophosphamide (i.v. CMF regimen, day 1, 21) as adjuvant chemotherapy for operable breast cancer: a study by the GEICAM group.
8683228	Sequential methotrexate and fluorouracil for the treatment of node-negative breast cancer patients with estrogen receptor-negative tumors: eight-year results from National Surgical Adjuvant Breast and Bowel Project (NSABP) B-13 and first report of findings from NSABP B-19 comparing methotrexate and fluorouracil with conventional cyclophosphamide, methotrexate, and fluorouracil.
12736280	High-dose chemotherapy with hematopoietic stem-cell rescue for multiple myeloma.
15925814	Gemcitabine versus cisplatin, epirubicin, fluorouracil, and gemcitabine in advanced pancreatic cancer: a randomised controlled multicentre phase III trial.
16467544	Radiotherapy plus cetuximab for squamous-cell carcinoma of the head and neck.
7715291	Efficacy of adjuvant fluorouracil and folinic acid in colon cancer. International Multicentre Pooled Analysis of Colon Cancer Trials (IMPACT) investigators.
7595708	Randomized comparison of vinorelbine and melphalan in anthracycline-refractory advanced breast cancer.
9890172	Effects of vinorelbine on quality of life and survival of elderly patients with advanced non-small-cell lung cancer. The Elderly Lung Cancer Vinorelbine Italian Study Group.
16750497	Immediate versus deferred androgen deprivation treatment in patients with node-positive prostate cancer after radical prostatectomy and pelvic lymphadenectomy.
21631324	gp100 peptide vaccine and interleukin-2 in patients with advanced melanoma.
23547078	Rituximab purging and/or maintenance in patients undergoing autologous transplantation for relapsed follicular lymphoma: a prospective randomized trial from the lymphoma working party of the European group for blood and marrow transplantation.

30613964	Concurrent chemoradiotherapy with/without induction chemotherapy in locoregionally advanced nasopharyngeal carcinoma: Long-term results of phase 3 randomized controlled trial.
10588962	Immediate hormonal therapy compared with observation after radical prostatectomy and pelvic lymphadenectomy in men with node-positive prostate cancer.
19339720	Cetuximab and chemotherapy as initial treatment for metastatic colorectal cancer.
22119496	Everolimus plus octreotide long-acting repeatable for the treatment of advanced neuroendocrine tumours associated with carcinoid syndrome (RADIANT-2): a randomised, placebo-controlled, phase 3 study.
27060152	DDGP versus SMILE in Newly Diagnosed Advanced Natural Killer/T-Cell Lymphoma: A Randomized Controlled, Multicenter, Open-label Study in China.
23569309	Phase III, multicenter, randomized trial of maintenance chemotherapy versus observation in patients with metastatic breast cancer after achieving disease control with six cycles of gemcitabine plus paclitaxel as first-line chemotherapy: KCSG-BR07-02.
25265492	Combined BRAF and MEK inhibition versus BRAF inhibition alone in melanoma.
30016391	Final results of a randomized phase III trial of induction chemotherapy followed by concurrent chemoradiotherapy versus concurrent chemoradiotherapy alone in patients with stage IVA and IVB nasopharyngeal carcinoma-Taiwan Cooperative Oncology Group (TCOG) 1303 Study.
11964279	Maintenance therapy with alternate-day prednisone improves survival in multiple myeloma patients.
12672279	A phase 3 trial of local chemotherapy with biodegradable carmustine (BCNU) wafers (Gliadel wafers) in patients with primary malignant glioma.
17693650	VAD-doxil versus VAD-doxil plus thalidomide as initial treatment for multiple myeloma: results of a multicenter randomized trial of the Greek Myeloma Study Group.
26884585	Randomized, Double-Blind, Placebo-Controlled Phase III Trial of Apatinib in Patients With Chemotherapy-Refractory Advanced or Metastatic Adenocarcinoma of the Stomach or Gastroesophageal Junction.
30199311	Efficacy and Tolerability of First-Line Cetuximab Plus Leucovorin, Fluorouracil, and Oxaliplatin (FOLFOX-4) Versus FOLFOX-4 in Patients With <i>RAS</i> Wild-Type Metastatic Colorectal Cancer: The Open-Label, Randomized, Phase III TAILOR Trial.
21751904	Radiotherapy and short-term androgen deprivation for localized prostate cancer.
22571201	Lenalidomide after stem-cell transplantation for multiple myeloma.
30842083	Long-term follow-up of the RESONATE phase 3 trial of ibrutinib vs ofatumumab.
20823406	Bortezomib plus dexamethasone is superior to vincristine plus doxorubicin plus dexamethasone as induction treatment prior to autologous stem-cell transplantation in newly diagnosed multiple myeloma: results of the IFM 2005-01 phase III trial.
1403038	A comparison of two short intensive adjuvant chemotherapy regimens in operable osteosarcoma of limbs in children and young adults: the first study of the European Osteosarcoma Intergroup.
21663857	Docetaxel vs. vinorelbine in elderly patients with advanced non--small-cell lung cancer: a hellenic oncology research group randomized phase III study.
10561343	Phase III study of concurrent versus sequential thoracic radiotherapy in combination with mitomycin, vindesine, and cisplatin in unresectable stage III non-small-cell lung cancer.

© 2019 Rubinstein SM et al. *JAMA Network Open.*

11483335	Phase III radiation therapy oncology group (RTOG) trial 86-10 of androgen deprivation adjuvant to definitive radiotherapy in locally advanced carcinoma of the prostate.
19410716	Cetuximab plus chemotherapy in patients with advanced non-small-cell lung cancer (FLEX): an open-label randomised phase III trial.
9632446	Hyperfractionated irradiation with or without concurrent chemotherapy for locally advanced head and neck cancer.
26927446	Capecitabine and bevacizumab with or without vinorelbine in first-line treatment of HER2/neu-negative metastatic or locally advanced breast cancer: final efficacy and safety data of the randomised, open-label superiority phase 3 CARIN trial.
17470860	Phase III trial of infusional fluorouracil, leucovorin, oxaliplatin, and irinotecan (FOLFOXIRI) compared with infusional fluorouracil, leucovorin, and irinotecan (FOLFIRI) as first-line treatment for metastatic colorectal cancer: the Gruppo Oncologico Nord Ovest.
22663011	Improved survival with MEK inhibition in BRAF-mutated melanoma.
25184862	Autologous transplantation and maintenance therapy in multiple myeloma.
30617130	Brentuximab Vedotin plus Chemotherapy in North American Subjects with Newly Diagnosed Stage III or IV Hodgkin Lymphoma.
24888810	Randomized, phase III trial of first-line figitumumab in combination with paclitaxel and carboplatin versus paclitaxel and carboplatin alone in patients with advanced non-small-cell lung cancer.
16419071	Phase III study of PSC-833 (valspodar) in combination with vincristine, doxorubicin, and dexamethasone (valspodar/VAD) versus VAD alone in patients with recurring or refractory multiple myeloma (E1A95): a trial of the Eastern Cooperative Oncology Group.
15385103	Long-term survival advantage for women treated with pegylated liposomal doxorubicin compared with topotecan in a phase 3 randomized study of recurrent and refractory epithelial ovarian cancer.
12377961	Adjuvant immunotherapy of patients with high-risk melanoma using vaccinia viral lysates of melanoma: results of a randomized trial.
16782917	Randomized phase III study of trastuzumab, paclitaxel, and carboplatin compared with trastuzumab and paclitaxel in women with HER-2-overexpressing metastatic breast cancer.
20818862	Sipuleucel-T immunotherapy for castration-resistant prostate cancer.
26410620	A randomized, controlled phase III trial of nab-Paclitaxel versus dacarbazine in chemotherapy-na√Øve patients with metastatic melanoma.
27718781	Adjuvant Sunitinib in High-Risk Renal-Cell Carcinoma after Nephrectomy.
15315996	6-month androgen suppression plus radiation therapy vs radiation therapy alone for patients with clinically localized prostate cancer: a randomized controlled trial.
21595951	Chemotherapy followed by surgery versus surgery alone in patients with resectable oesophageal squamous cell carcinoma: long-term results of a randomized controlled trial.
24317176	Final overall survival: fulvestrant 500 mg vs 250 mg in the randomized CONFIRM trial.
29533000	Ibrutinib versus rituximab in relapsed or refractory chronic lymphocytic leukemia or small lymphocytic lymphoma: a randomized, open-label phase 3 study.
12944571	Neoadjuvant chemotherapy plus cystectomy compared with cystectomy alone for locally advanced bladder cancer.
19933916	Long-term results of the international adjuvant lung cancer trial evaluating adjuvant Cisplatin-based chemotherapy in resected lung cancer.

© 2019 Rubinstein SM et al. *JAMA Network Open.*

19690954	Randomized phase III trial of trastuzumab monotherapy followed by trastuzumab plus docetaxel versus trastuzumab plus docetaxel as first-line therapy in patients with HER2-positive metastatic breast cancer: the JO17360 Trial Group.
21742485	Survival advantage for irinotecan versus best supportive care as second-line chemotherapy in gastric cancer--a randomised phase III study of the Arbeitsgemeinschaft Internistische Onkologie (AIO).
25559811	Phase III Trial to Compare Adjuvant Chemotherapy With Capecitabine and Cisplatin Versus Concurrent Chemoradiotherapy in Gastric Cancer: Final Report of the Adjuvant Chemoradiotherapy in Stomach Tumors Trial, Including Survival and Subset Analyses.
24127442	Sequential combination of gemtuzumab ozogamicin and standard chemotherapy in older patients with newly diagnosed acute myeloid leukemia: results of a randomized phase III trial by the EORTC and GIMEMA consortium (AML-17).
12881382	Adjuvant therapy after excision and radiation of isolated postmastectomy locoregional breast cancer recurrence: definitive results of a phase III randomized trial (SAKK 23/82) comparing tamoxifen with observation.
12226148	A randomized trial comparing radical prostatectomy with watchful waiting in early prostate cancer.
25515658	Randomized phase III trial of prophylactic cranial irradiation versus observation in patients with fully resected stage IIIA-N2 nonsmall-cell lung cancer and high risk of cerebral metastases after adjuvant chemotherapy.
11230478	Results of a prospective randomized trial comparing neoadjuvant chemotherapy plus radiotherapy with radiotherapy alone in patients with locoregionally advanced nasopharyngeal carcinoma.
10674879	Phase III trial of cyclophosphamide, epirubicin, fluorouracil (CEF) versus cyclophosphamide, mitoxantrone, fluorouracil (CNF) in women with metastatic breast cancer.
21730266	Tandem autologous/reduced-intensity conditioning allogeneic stem-cell transplantation versus autologous transplantation in myeloma: long-term follow-up.
23233710	Phase III randomized intergroup trial of CHOP plus rituximab compared with CHOP chemotherapy plus (131)iodine-tositumomab for previously untreated follicular non-Hodgkin lymphoma: SWOG S0016.
27987591	Nintedanib plus pemetrexed versus placebo plus pemetrexed in patients with relapsed or refractory, advanced non-small cell lung cancer (LUME-Lung 2): A randomized, double-blind, phase III trial.
11806971	Comparison of 200 mg/m(2) melphalan and 8 Gy total body irradiation plus 140 mg/m(2) melphalan as conditioning regimens for peripheral blood stem cell transplantation in patients with newly diagnosed multiple myeloma: final analysis of the Intergroupe Francophone du My√©lome 9502 randomized trial.
11697832	Clinical-benefit response in advanced non-small-cell lung cancer: A multicentre prospective randomised phase III study of single agent gemcitabine versus cisplatin-vindesine.
15700852	Gemcitabine versus FLEC regimen given intra-arterially to patients with unresectable pancreatic cancer: a prospective, randomized phase III trial of the Italian Society for Integrated Locoregional Therapy in Oncology.
1302472	Treatment of advanced squamous-cell carcinoma of the head and neck with alternating chemotherapy and radiotherapy.
19451431	Improved overall survival with oxaliplatin, fluorouracil, and leucovorin as adjuvant treatment in stage II or III colon cancer in the MOSAIC trial.
19933915	Randomized phase III trial of vinorelbine plus cisplatin compared with observation in completely resected stage IB and II non-small-cell lung cancer: updated survival analysis of JBR-10.

© 2019 Rubinstein SM et al. *JAMA Network Open.*

30305149	Adjuvant transcatheter arterial chemoembolization after curative resection for hepatocellular carcinoma patients with solitary tumor and microvascular invasion: a randomized clinical trial of efficacy and safety.
20082144	Long-term survival results of surgery alone versus surgery plus 5-fluorouracil and leucovorin for stage II and stage III colon cancer: pooled analysis of NSABP C-01 through C-05. A baseline from which to compare modern adjuvant trials.
12598351	Doxorubicin versus doxorubicin and cisplatin in endometrial carcinoma: definitive results of a randomised study (55872) by the EORTC Gynaecological Cancer Group.
9440721	Maintenance of remission with human recombinant interferon alfa-2a in patients with stages III and IV low-grade malignant non-Hodgkin's lymphoma. European Organization for Research and Treatment of Cancer Lymphoma Cooperative Group.
23091096	Randomized controlled trial of interval-compressed chemotherapy for the treatment of localized Ewing sarcoma: a report from the Children's Oncology Group.
16293869	Phase III study of second-line chemotherapy for advanced non-small-cell lung cancer with weekly compared with 3-weekly docetaxel.
8872324	Advanced breast cancer: a randomized study of doxorubicin or mitoxantrone in combination with cyclophosphamide and vincristine.
1607916	A phase III trial comparing idarubicin and daunorubicin in combination with cytarabine in acute myelogenous leukemia: a Southeastern Cancer Study Group Study.
17135639	Phase III trial comparing doxorubicin plus cyclophosphamide with docetaxel plus cyclophosphamide as adjuvant therapy for operable breast cancer.
9196130	Topotecan versus paclitaxel for the treatment of recurrent epithelial ovarian cancer.
8040675	Tamoxifen versus high-dose oral medroxyprogesterone acetate as initial endocrine therapy for patients with metastatic breast cancer: a Piedmont Oncology Association study.
2475589	On the value of intensive remission-induction chemotherapy in elderly patients of 65+ years with acute myeloid leukemia: a randomized phase III study of the European Organization for Research and Treatment of Cancer Leukemia Group.
16945766	Adjuvant vinorelbine plus cisplatin versus observation in patients with completely resected stage IB-IIIA non-small-cell lung cancer (Adjuvant Navelbine International Trialist Association [ANITA]): a randomised controlled trial.
16330205	Seven year update of an EORTC phase III trial of high-dose intensity M-VAC chemotherapy and G-CSF versus classic M-VAC in advanced urothelial tract tumours.
19204201	Docetaxel With Cyclophosphamide Is Associated With an Overall Survival Benefit Compared With Doxorubicin and Cyclophosphamide: 7-Year Follow-Up of US Oncology Research Trial 9735.
19770376	Preoperative multimodality therapy improves disease-free survival in patients with carcinoma of the rectum: NSABP R-03.
9740079	Anastrozole versus megestrol acetate in the treatment of postmenopausal women with advanced breast carcinoma: results of a survival update based on a combined analysis of data from two mature phase III trials. Arimidex Study Group.
18073378	Extended adjuvant therapy with anastrozole among postmenopausal breast cancer patients: results from the randomized Austrian Breast and Colorectal Cancer Study Group Trial 6a.
21959045	Efficacy and safety of bevacizumab plus chemotherapy in Chinese patients with metastatic colorectal cancer: a randomized phase III ARTIST trial.

© 2019 Rubinstein SM et al. *JAMA Network Open.*

22689805	Multicenter, randomized, open-label, phase III trial of decitabine versus patient choice, with physician advice, of either supportive care or low-dose cytarabine for the treatment of older patients with newly diagnosed acute myeloid leukemia.
19451428	Efficacy of melphalan and prednisone plus thalidomide in patients older than 75 years with newly diagnosed multiple myeloma: IFM 01/01 trial.
22541893	Trabectedin plus pegylated liposomal doxorubicin (PLD) versus PLD in recurrent ovarian cancer: overall survival analysis.
9643663	A randomised comparison of 'Casodex' (bicalutamide) 150 mg monotherapy versus castration in the treatment of metastatic and locally advanced prostate cancer.
16192591	Phase II to III study comparing doxorubicin and docetaxel with fluorouracil, doxorubicin, and cyclophosphamide as first-line chemotherapy in patients with metastatic breast cancer: results of a Dutch Community Setting Trial for the Clinical Trial Group of the Comprehensive Cancer Centre.
18669453	Randomized phase III study comparing preoperative radiotherapy with chemoradiotherapy in nonresectable rectal cancer.
2649221	Randomized clinical trial of CFP versus CMFP in women with metastatic breast cancer.
19364964	Intensive dose-dense compared with conventionally scheduled preoperative chemotherapy for high-risk primary breast cancer.
22493375	Intra-arterial infusion of irinotecan-loaded drug-eluting beads (DEBIRI) versus intravenous therapy (FOLFIRI) for hepatic metastases from colorectal cancer: final results of a phase III study.
29190507	Results of methotrexate-etoposide-ifosfamide based regimen (M-EI) in osteosarcoma patients included in the French OS2006/sarcome-09 study.
18281662	Phase III trial of gemcitabine compared with pegylated liposomal doxorubicin in progressive or recurrent ovarian cancer.
16782910	Phase III trial of chemotherapy plus radiotherapy compared with radiotherapy alone for pure and mixed anaplastic oligodendroglioma: Intergroup Radiation Therapy Oncology Group Trial 9402.
7595712	Cisplatin plus etoposide with and without ifosfamide in extensive small-cell lung cancer: a Hoosier Oncology Group study.
17135646	Phase III trial comparing supportive care alone with supportive care with oral topotecan in patients with relapsed small-cell lung cancer.
17855669	High-dose melphalan versus melphalan plus dexamethasone for AL amyloidosis.
25925990	Phase III randomized trial of second-line ixabepilone versus paclitaxel or doxorubicin in women with advanced endometrial cancer.
30017831	Nedaplatin Plus Docetaxel Versus Cisplatin Plus Docetaxel as First-Line Chemotherapy for Advanced Squamous Cell Carcinoma of the Lung - A Multicenter, Open-label, Randomized, Phase III Trial.
23980077	Randomized, multicenter, open-label study of oxaliplatin plus fluorouracil/leucovorin versus doxorubicin as palliative chemotherapy in patients with advanced hepatocellular carcinoma from Asia.
19075278	Phase III study of immediate compared with delayed docetaxel after front-line therapy with gemcitabine plus carboplatin in advanced non-small-cell lung cancer.
16192580	Randomized phase III study of cisplatin with or without raltitrexed in patients with malignant pleural mesothelioma: an intergroup study of the European Organisation for Research and Treatment of Cancer Lung Cancer Group and the National Cancer Institute of Canada.
24449241	Bortezomib-melphalan-prednisone-thalidomide followed by maintenance with bortezomib-thalidomide compared with bortezomib-melphalan-prednisone for initial treatment of multiple myeloma: updated follow-up and improved survival.

22084374	Gemcitabine plus docetaxel versus docetaxel in patients with predominantly human epidermal growth factor receptor 2-negative locally advanced or metastatic breast cancer: a randomized, phase III study by the Danish Breast Cancer Cooperative Group.
11870165	Randomized controlled trial of single-agent paclitaxel versus cyclophosphamide, doxorubicin, and cisplatin in patients with recurrent ovarian cancer who responded to first-line platinum-based regimens.
14665607	Docetaxel and cisplatin with granulocyte colony-stimulating factor (G-CSF) versus MVAC with G-CSF in advanced urothelial carcinoma: a multicenter, randomized, phase III study from the Hellenic Cooperative Oncology Group.
19636702	FEC versus sequential docetaxel followed by epirubicin/cyclophosphamide as adjuvant chemotherapy in women with axillary node-positive early breast cancer: a randomized study of the Hellenic Oncology Research Group (HORG).
20879881	Anti-GD2 antibody with GM-CSF, interleukin-2, and isotretinoin for neuroblastoma.
20516432	Trabectedin plus pegylated liposomal Doxorubicin in recurrent ovarian cancer.
24024839	Panitumumab-FOLFOX4 treatment and RAS mutations in colorectal cancer.
20516432	Trabectedin plus pegylated liposomal Doxorubicin in recurrent ovarian cancer.
16921044	Low locoregional recurrence rate among node-negative breast cancer patients with tumors 5 cm or larger treated by mastectomy, with or without adjuvant systemic therapy and without radiotherapy: results from five national surgical adjuvant breast and bowel project randomized clinical trials.
16921044	Low locoregional recurrence rate among node-negative breast cancer patients with tumors 5 cm or larger treated by mastectomy, with or without adjuvant systemic therapy and without radiotherapy: results from five national surgical adjuvant breast and bowel project randomized clinical trials.
11579113	Randomized clinical trial of adjuvant fluorouracil, epirubicin, and cyclophosphamide chemotherapy for patients with fast-proliferating, node-negative breast cancer.
17308271	Double-blinded randomized study of high-dose calcitriol plus docetaxel compared with placebo plus docetaxel in androgen-independent prostate cancer: a report from the ASCENT Investigators.
22124104	Randomized phase III study of surgery alone or surgery plus preoperative cisplatin and gemcitabine in stages IB to IIIA non-small-cell lung cancer.
23769990	Fludarabine and 2-Gy TBI is superior to 2 Gy TBI as conditioning for HLA-matched related hematopoietic cell transplantation: a phase III randomized trial.
8839901	First-line fadrozole HCI (CGS 16949A) versus tamoxifen in postmenopausal women with advanced breast cancer. Prospective randomised trial of the Swiss Group for Clinical Cancer Research SAKK 20/88.
7595735	Final results of a phase III clinical trial of adjuvant chemotherapy with the modified fluorouracil, doxorubicin, and mitomycin regimen in resectable gastric cancer.
2455701	Combined modality treatment for stage I-II non-Hodgkin's lymphomas: CVP versus BACOP chemotherapy.
9227927	Interferon alfa-2b combined with cytarabine versus interferon alone in chronic myelogenous leukemia. French Chronic Myeloid Leukemia Study Group.
16087943	Weekly paclitaxel improves pathologic complete remission in operable breast cancer when compared with paclitaxel once every 3 weeks.
19414675	Placebo-controlled phase III trial of patient-specific immunotherapy with mitumprotimut-T and granulocyte-macrophage colony-stimulating factor after rituximab in patients with follicular lymphoma.
21251813	Weekly docetaxel in metastatic breast cancer patients: no superior benefits compared to three-weekly docetaxel.

© 2019 Rubinstein SM et al. *JAMA Network Open.*

23733779	Fluorouracil, doxorubicin, and cyclophosphamide (FAC) versus FAC followed by weekly paclitaxel as adjuvant therapy for high-risk, node-negative breast cancer: results from the GEICAM/2003-02 study.
15625368	Randomized phase III trial of high-dose interleukin-2 versus subcutaneous interleukin-2 and interferon in patients with metastatic renal cell carcinoma.
8558223	Interferon alfa-2b adjuvant therapy of high-risk resected cutaneous melanoma: the Eastern Cooperative Oncology Group Trial EST 1684.
12637459	Docetaxel and doxorubicin compared with doxorubicin and cyclophosphamide as first-line chemotherapy for metastatic breast cancer: results of a randomized, multicenter, phase III trial.
20888709	Phase III study of radiation therapy with or without cis-platinum in patients with unresectable squamous or undifferentiated carcinoma of the head and neck: an intergroup trial of the Eastern Cooperative Oncology Group (E2382).
16260697	Temozolomide in combination with interferon-alfa versus temozolomide alone in patients with advanced metastatic melanoma: a randomized, phase III, multicenter study from the Dermatologic Cooperative Oncology Group.
17290061	Phase III trial of ifosfamide with or without paclitaxel in advanced uterine carcinosarcoma: a Gynecologic Oncology Group Study.
17047649	Weekly cisplatin, epirubicin, and paclitaxel with granulocyte colony-stimulating factor support vs triweekly epirubicin and paclitaxel in locally advanced breast cancer: final analysis of a sicog phase III study.
21444866	Perioperative chemotherapy compared with surgery alone for resectable gastroesophageal adenocarcinoma: an FNCLCC and FFCD multicenter phase III trial.
22689807	Overall survival benefit with lapatinib in combination with trastuzumab for patients with human epidermal growth factor receptor 2-positive metastatic breast cancer: final results from the EGF104900 Study.
2462025	Vinblastine, bleomycin, and methotrexate: an effective adjuvant in favorable Hodgkin's disease.
17031690	High-vs low-dose cytarabine combined with interferon alfa in patients with first chronic phase chronic myeloid leukemia. A prospective randomized phase III study.
21741829	Trastuzumab beyond progression: overall survival analysis of the GBG 26/BIG 3-05 phase III study in HER2-positive breast cancer.
24560487	Biweekly irinotecan plus cisplatin versus irinotecan alone as second-line treatment for advanced gastric cancer: a randomised phase III trial (TCOG GI-0801/BIRIP trial).
18445839	Individual fluorouracil dose adjustment based on pharmacokinetic follow-up compared with conventional dosage: results of a multicenter randomized trial of patients with metastatic colorectal cancer.
2451713	Sequential versus alternating chemotherapy and radiotherapy in stage III-IV squamous cell carcinoma of the head and neck: a phase III study.
7845115	Long-term results of single course of adjuvant intraportal chemotherapy for colorectal cancer. Swiss Group for Clinical Cancer Research (SAKK)
7595704	Comparison of doxorubicin and mitoxantrone in the treatment of elderly patients with advanced diffuse non-Hodgkin's lymphoma using CHOP versus CNOP chemotherapy.
7477169	Autologous bone marrow transplantation as compared with salvage chemotherapy in relapses of chemotherapy-sensitive non-Hodgkin's lymphoma.
24395447	Tamoxifen versus tamoxifen plus doxorubicin and cyclophosphamide as adjuvant therapy for node-positive postmenopausal breast cancer: results of a Japan Clinical Oncology Group Study (JCOG9401).
26763986	Reduced intensity VEPEMB regimen compared with standard ABVD in elderly Hodgkin lymphoma patients: results from a randomized trial on behalf of the Fondazione Italiana Linfomi (FIL).

© 2019 Rubinstein SM et al. *JAMA Network Open.*

20530281	Phase III trial comparing docetaxel and cisplatin combination chemotherapy with mitomycin, vindesine, and cisplatin combination chemotherapy with concurrent thoracic radiotherapy in locally advanced non-small-cell lung cancer: OLCSG 0007.
8386751	Randomized trial of etoposide and cisplatin versus etoposide and carboplatin in patients with good-risk germ cell tumors: a multiinstitutional study.
8247036	Initial chemotherapeutic doses and survival in patients with limited small-cell lung cancer.
9647875	Chemotherapy alone compared with chemotherapy plus radiotherapy for localized intermediate- and high-grade non-Hodgkin's lymphoma.
12089229	ChlVPP/EVA hybrid versus the weekly VAPEC-B regimen for previously untreated Hodgkin's disease.
15718308	Hyperfractionated accelerated chemoradiation with concurrent fluorouracil-mitomycin is more effective than dose-escalated hyperfractionated accelerated radiation therapy alone in locally advanced head and neck cancer: final results of the radiotherapy cooperative clinical trials group of the German Cancer Society 95-06 Prospective Randomized Trial.
17116941	Sequential adjuvant epirubicin-based and docetaxel chemotherapy for node-positive breast cancer patients: the FNCLCC PACS 01 Trial.
17538164	Quality of life in head and neck cancer patients after treatment with high-dose radiotherapy alone or in combination with cetuximab.
21474675	Effectiveness of high-dose methotrexate in T-cell lymphoblastic leukemia and advanced-stage lymphoblastic lymphoma: a randomized study by the Children's Oncology Group (POG 9404).
24019545	Tivozanib versus sorafenib as initial targeted therapy for patients with metastatic renal cell carcinoma: results from a phase III trial.
24841974	Randomized phase III trial of erlotinib versus docetaxel as second- or third-line therapy in patients with advanced non-small-cell lung cancer: Docetaxel and Erlotinib Lung Cancer Trial (DELTA).
11251000	Doxorubicin and paclitaxel versus fluorouracil, doxorubicin, and cyclophosphamide as first-line therapy for women with metastatic breast cancer: final results of a randomized phase III multicenter trial.
24347519	Epirubicin and docetaxel with or without capecitabine as neoadjuvant treatment for early breast cancer: final results of a randomized phase III study (ABCSG-24).
8961989	Randomised trial of surgery alone versus radiotherapy followed by surgery for potentially operable locally advanced rectal cancer. Medical Research Council Rectal Cancer Working Party.
10880550	Randomized trial of paclitaxel plus supportive care versus supportive care for patients with advanced non-small-cell lung cancer.
10623706	Randomized phase III study of temozolomide versus dacarbazine in the treatment of patients with advanced metastatic malignant melanoma.
12529345	International Collaborative Ovarian Neoplasm trial 1: a randomized trial of adjuvant chemotherapy in women with early-stage ovarian cancer.
15459211	Phase III trial of doxorubicin with or without cisplatin in advanced endometrial carcinoma: a gynecologic oncology group study.
15020614	Fotemustine compared with dacarbazine in patients with disseminated malignant melanoma: a phase III study.
17296974	Randomized phase III trial of fludarabine plus cyclophosphamide with or without oblimersen sodium (Bcl-2 antisense) in patients with relapsed or refractory chronic lymphocytic leukemia.
21969502	Gemcitabine alone versus gemcitabine plus radiotherapy in patients with locally advanced pancreatic cancer: an Eastern Cooperative Oncology Group trial.
25482145	Carfilzomib, lenalidomide, and dexamethasone for relapsed multiple myeloma.

© 2019 Rubinstein SM et al. *JAMA Network Open.*

11956263	Concurrent chemotherapy-radiotherapy compared with radiotherapy alone in locoregionally advanced nasopharyngeal carcinoma: progression-free survival analysis of a phase III randomized trial.
12837810	Gemcitabine plus vinorelbine compared with cisplatin plus vinorelbine or cisplatin plus gemcitabine for advanced non-small-cell lung cancer: a phase III trial of the Italian GEMVIN Investigators and the National Cancer Institute of Canada Clinical Trials Group.
12663707	Phase III study of interferon alfa-NL as adjuvant treatment for resectable renal cell carcinoma: an Eastern Cooperative Oncology Group/Intergroup trial.
8367920	Goserelin acetate and flutamide versus bilateral orchiectomy: a phase III EORTC trial (30853). EORTC GU Group and EORTC Data Center.
15908661	Gemcitabine in combination with oxaliplatin compared with gemcitabine alone in locally advanced or metastatic pancreatic cancer: results of a GERCOR and GISCAD phase III trial.
15657403	Long-term survival after cisplatin-based induction chemotherapy and radiotherapy for nasopharyngeal carcinoma: a pooled data analysis of two phase III trials.
20194844	Rituximab plus fludarabine and cyclophosphamide prolongs progression-free survival compared with fludarabine and cyclophosphamide alone in previously treated chronic lymphocytic leukemia.
15657403	Long-term survival after cisplatin-based induction chemotherapy and radiotherapy for nasopharyngeal carcinoma: a pooled data analysis of two phase III trials.
16754935	Rituximab-CHOP versus CHOP alone or with maintenance rituximab in older patients with diffuse large B-cell lymphoma.
11863118	Maintenance daily oral etoposide versus no further therapy following induction chemotherapy with etoposide plus ifosfamide plus cisplatin in extensive small-cell lung cancer: a Hoosier Oncology Group randomized study.
11454878	Recurrent epithelial ovarian carcinoma: a randomized phase III study of pegylated liposomal doxorubicin versus topotecan.
10506613	Vinblastine versus vinblastine plus oral estramustine phosphate for patients with hormone-refractory prostate cancer: A Hoosier Oncology Group and Fox Chase Network phase III trial.
17452677	Erlotinib plus gemcitabine compared with gemcitabine alone in patients with advanced pancreatic cancer: a phase III trial of the National Cancer Institute of Canada Clinical Trials Group.
16809734	Placebo-controlled phase III trial of immunologic therapy with sipuleucel-T (APC8015) in patients with metastatic, asymptomatic hormone refractory prostate cancer.
15117982	Comparison of fulvestrant versus tamoxifen for the treatment of advanced breast cancer in postmenopausal women previously untreated with endocrine therapy: a multinational, double-blind, randomized trial.
8232429	Recombinant interferon alfa-2b combined with a regimen containing doxorubicin in patients with advanced follicular lymphoma. Groupe d'Etude des Lymphomes de l'Adulte.
14722033	Phase III randomized trial of docetaxel plus cisplatin versus vindesine plus cisplatin in patients with stage IV non-small-cell lung cancer: the Japanese Taxotere Lung Cancer Study Group.
17563395	Improved overall survival in postmenopausal women with early breast cancer after anastrozole initiated after treatment with tamoxifen compared with continued tamoxifen: the ARNO 95 Study.
12153376	Comparison of low-molecular-weight heparin and warfarin for the secondary prevention of venous thromboembolism in patients with cancer: a randomized controlled study.

© 2019 Rubinstein SM et al. *JAMA Network Open.*

10623704	Phase III multicenter randomized trial of oxaliplatin added to chronomodulated fluorouracil-leucovorin as first-line treatment of metastatic colorectal cancer.
19770374	Long-term results of a randomized trial of surgery with or without preoperative chemotherapy in esophageal cancer.
22370319	Randomized phase III study comparing paclitaxel/cisplatin/gemcitabine and gemcitabine/cisplatin in patients with locally advanced or metastatic urothelial cancer without prior systemic therapy: EORTC Intergroup Study 30987.
14987883	Adjuvant autologous renal tumour cell vaccine and risk of tumour progression in patients with renal-cell carcinoma after radical nephrectomy: phase III, randomised controlled trial.
14657228	Final results of the 94-01 French Head and Neck Oncology and Radiotherapy Group randomized trial comparing radiotherapy alone with concomitant radiochemotherapy in advanced-stage oropharynx carcinoma.
19289619	Trastuzumab beyond progression in human epidermal growth factor receptor 2-positive advanced breast cancer: a german breast group 26/breast international group 03-05 study.
21828126	A randomized trial comparing standard versus high-dose daunorubicin induction in patients with acute myeloid leukemia.
16921047	Randomized phase III trial of gemcitabine plus cisplatin compared with gemcitabine alone in advanced pancreatic cancer.
7526096	Quality of life and survival with continuous hepatic-artery floxuridine infusion for colorectal liver metastases.
9654256	Randomised trial of interferon alpha-2a as adjuvant therapy in resected primary melanoma thicker than 1.5 mm without clinically detectable node metastases. French Cooperative Group on Melanoma.
23482933	Autologous/reduced-intensity allogeneic stem cell transplantation vs autologous transplantation in multiple myeloma: long-term results of the EBMT-NMAM2000 study.
24450857	Idelalisib and rituximab in relapsed chronic lymphocytic leukemia.
30729512	The impact of cytogenetics on duration of response and overall survival in patients with relapsed multiple myeloma (long-term follow-up results from BSBMT/UKMF Myeloma X Relapse [Intensive]): a randomised, open-label, phase 3 trial.
9654256	Randomised trial of interferon alpha-2a as adjuvant therapy in resected primary melanoma thicker than 1.5 mm without clinically detectable node metastases. French Cooperative Group on Melanoma.
10550150	Gemcitabine and cisplatin versus mitomycin, ifosfamide, and cisplatin in advanced non-small-cell lung cancer: A randomized phase III study of the Italian Lung Cancer Project.
27626518	Rituximab in B-Lineage Adult Acute Lymphoblastic Leukemia.
17968021	VCAP-AMP-VECP compared with biweekly CHOP for adult T-cell leukemia-lymphoma: Japan Clinical Oncology Group Study JCOG9801.
18172188	Short-term neoadjuvant androgen deprivation therapy and external-beam radiotherapy for locally advanced prostate cancer: long-term results of RTOG 8610.
10942228	Low-dose cytarabine maintenance therapy vs observation after remission induction in advanced acute myeloid leukemia: an Eastern Cooperative Oncology Group Trial (E5483).
15284112	The addition of rituximab to a combination of fludarabine, cyclophosphamide, mitoxantrone (FCM) significantly increases the response rate and prolongs survival as compared with FCM alone in patients with relapsed and refractory follicular and mantle cell lymphomas: results of a prospective randomized study of the German Low-Grade Lymphoma Study Group.

© 2019 Rubinstein SM et al. *JAMA Network Open.*

21263100	Phase III trial of weekly methotrexate or pulsed dactinomycin for low-risk gestational trophoblastic neoplasia: a gynecologic oncology group study.
12118018	Phase III study of concurrent versus sequential thoracic radiotherapy in combination with cisplatin and etoposide for limited-stage small-cell lung cancer: results of the Japan Clinical Oncology Group Study 9104.
10561349	Phase III multicenter randomized trial of the Dartmouth regimen versus dacarbazine in patients with metastatic melanoma.
10577853	Randomized clinical trial of adjuvant mitomycin plus tegafur in patients with resected stage III gastric cancer.
10334517	Randomized comparison of fluorouracil plus cisplatin versus hydroxyurea as an adjunct to radiation therapy in stage IIB-IVA carcinoma of the cervix with negative para-aortic lymph nodes: a Gynecologic Oncology Group and Southwest Oncology Group study.
10735887	Exemestane is superior to megestrol acetate after tamoxifen failure in postmenopausal women with advanced breast cancer: results of a phase III randomized double-blind trial. The Exemestane Study Group.
15102997	A randomized trial of adjuvant chemotherapy with uracil-tegafur for adenocarcinoma of the lung.
14976046	Prolonged treatment with rituximab in patients with follicular lymphoma significantly increases event-free survival and response duration compared with the standard weekly x 4 schedule.
18711184	Gemcitabine plus Paclitaxel versus Paclitaxel monotherapy in patients with metastatic breast cancer and prior anthracycline treatment.
9440718	MOPP/ABV hybrid chemotherapy for advanced Hodgkin's disease significantly improves failure-free and overall survival: the 8-year results of the intergroup trial.
12441935	Phase III study of mitoxantrone plus low dose prednisone versus low dose prednisone alone in patients with asymptomatic hormone refractory prostate cancer.
17075117	Phase III study of docetaxel and cisplatin plus fluorouracil compared with cisplatin and fluorouracil as first-line therapy for advanced gastric cancer: a report of the V325 Study Group.
17317858	Drug treatment is superior to allografting as first-line therapy in chronic myeloid leukemia.
19001327	Phase III trial comparing concurrent biochemotherapy with cisplatin, vinblastine, dacarbazine, interleukin-2, and interferon alfa-2b with cisplatin, vinblastine, and dacarbazine alone in patients with metastatic malignant melanoma (E3695): a trial coordinated by the Eastern Cooperative Oncology Group.
28463149	Duration of Androgen Deprivation in Locally Advanced Prostate Cancer: Long-Term Update of NRG Oncology RTOG 9202.
21951683	Autologous peripheral blood stem cell transplantation for acute myeloid leukemia.
11352955	Randomized phase III trial of high-dose-intensity methotrexate, vinblastine, doxorubicin, and cisplatin (MVAC) chemotherapy and recombinant human granulocyte colony-stimulating factor versus classic MVAC in advanced urothelial tract tumors: European Organization for Research and Treatment of Cancer Protocol no. 30924.
12920037	Intensive conventional chemotherapy (ACVBP regimen) compared with standard CHOP for poor-prognosis aggressive non-Hodgkin lymphoma.
15169803	Phase III trial of doxorubicin plus cisplatin with or without paclitaxel plus filgrastim in advanced endometrial carcinoma: a Gynecologic Oncology Group Study.
17341661	Postremission treatment of elderly patients with acute myeloid leukemia in first complete remission after intensive induction chemotherapy: results of the multicenter randomized Acute Leukemia French Association (ALFA) 9803 trial.

© 2019 Rubinstein SM et al. *JAMA Network Open.*

16682727	Phase III study comparing oral topotecan to intravenous docetaxel in patients with pretreated advanced non-small-cell lung cancer.
15351193	Treatment of lymph-node-negative, oestrogen-receptor-positive breast cancer: long-term findings from National Surgical Adjuvant Breast and Bowel Project randomised clinical trials.
15315964	Results of a prospective randomized clinical trial of doxorubicin, bleomycin, vinblastine, and dacarbazine (ABVD) followed by radiation therapy (RT) versus ABVD alone for stages I, II, and IIIA nonbulky Hodgkin disease.
10637238	Comparison of chemotherapy with chemohormonal therapy as first-line therapy for metastatic, hormone-sensitive breast cancer: An Eastern Cooperative Oncology Group study.
12860938	Phase III study of pemetrexed in combination with cisplatin versus cisplatin alone in patients with malignant pleural mesothelioma.
15044639	Initial treatment of aggressive lymphoma with high-dose chemotherapy and autologous stem-cell support.
16484584	Fludarabine and cytosine are less effective than standard ADE chemotherapy in high-risk acute myeloid leukemia, and addition of G-CSF and ATRA are not beneficial: results of the MRC AML-HR randomized trial.
20368558	Phase III trial of bevacizumab plus interferon alfa versus interferon alfa monotherapy in patients with metastatic renal cell carcinoma: final results of CALGB 90206.
9807986	Randomised trial of irinotecan versus fluorouracil by continuous infusion after fluorouracil failure in patients with metastatic colorectal cancer.
16293868	Phase III trial of gemcitabine plus carboplatin versus single-agent gemcitabine in the treatment of locally advanced or metastatic non-small-cell lung cancer: the Swedish Lung Cancer Study Group.
18048644	In adults with standard-risk acute lymphoblastic leukemia, the greatest benefit is achieved from a matched sibling allogeneic transplantation in first complete remission, and an autologous transplantation is less effective than conventional consolidation/maintenance chemotherapy in all patients: final results of the International ALL Trial (MRC UKALL XII/ECOG E2993).
20145166	Randomized Phase III trial of gefitinib versus docetaxel in non-small cell lung cancer patients who have previously received platinum-based chemotherapy.
24415640	Chlorambucil plus rituximab with or without maintenance rituximab as first-line treatment for elderly chronic lymphocytic leukemia patients.
15197192	Phase III study of intravenous vinorelbine in combination with epirubicin versus epirubicin alone in patients with advanced breast cancer: a Scandinavian Breast Group Trial (SBG9403).
21879261	A randomized trial comparing postoperative adjuvant chemotherapy with cisplatin and 5-fluorouracil versus preoperative chemotherapy for localized advanced squamous cell carcinoma of the thoracic esophagus (JCOG9907).
24356622	Final results from a randomized phase 3 study of FOLFIRI {+/-} panitumumab for second-line treatment of metastatic colorectal cancer.
12826431	Paclitaxel plus platinum-based chemotherapy versus conventional platinum-based chemotherapy in women with relapsed ovarian cancer: the ICON4/AGO-OVAR-2.2 trial.
15210738	Chemotherapy with or without radiotherapy in limited-stage diffuse aggressive non-Hodgkin's lymphoma: Eastern Cooperative Oncology Group study 1484.
17075115	Multicenter randomized trial of adjuvant fluorouracil and folinic acid compared with surgery alone after resection of colorectal liver metastases: FFCD ACHBTH AURC 9002 trial.
23109696	Clinical outcome of the ACCORD 12/0405 PRODIGE 2 randomized trial in rectal cancer.

© 2019 Rubinstein SM et al. *JAMA Network Open.*

17075115	Multicenter randomized trial of adjuvant fluorouracil and folinic acid compared with surgery alone after resection of colorectal liver metastases: FFCD ACHBTH AURC 9002 trial.
9653495	The impact of adding low-dose leucovorin to monthly 5-fluorouracil in advanced colorectal carcinoma: results of a phase III trial. Swiss Group for Clinical Cancer Research (SAKK).
14551293	Randomized trial of cytoreduction and hyperthermic intraperitoneal chemotherapy versus systemic chemotherapy and palliative surgery in patients with peritoneal carcinomatosis of colorectal cancer.
15726120	Cisplatin plus oral etoposide (EoP) combination is more effective than paclitaxel in patients with advanced breast cancer pretreated with anthracyclines: a randomised phase III trial of Turkish Oncology Group.
20458045	Intense dose-dense sequential chemotherapy with epirubicin, paclitaxel, and cyclophosphamide compared with conventionally scheduled chemotherapy in high-risk primary breast cancer: mature results of an AGO phase III study.
16257339	Gefitinib plus best supportive care in previously treated patients with refractory advanced non-small-cell lung cancer: results from a randomised, placebo-controlled, multicentre study (Iressa Survival Evaluation in Lung Cancer).
14679127	Long-term survival in a phase III, randomised study of topotecan versus paclitaxel in advanced epithelial ovarian carcinoma.
7799039	Ten-year results of a randomized trial evaluating prolonged low-dose adjuvant chemotherapy in node-positive breast cancer: a joint European Organization for Research and Treatment of Cancer-Dutch Breast Cancer Working Party Study. Cooperating Investigators.
9920950	Twice-daily compared with once-daily thoracic radiotherapy in limited small-cell lung cancer treated concurrently with cisplatin and etoposide.
8280653	Primary endocrine therapy for advanced breast cancer: to start with tamoxifen or with medroxyprogesterone acetate?
15451219	Progression-free survival in gastrointestinal stromal tumours with high-dose imatinib: randomised trial.
27002117	VTD is superior to VCD prior to intensive therapy in multiple myeloma: results of the prospective IFM2013-04 trial.
9552047	Adjuvant interferon alfa-2a treatment in resected primary stage II cutaneous melanoma. Austrian Malignant Melanoma Cooperative Group.
16000354	Hydroxyurea compared with anagrelide in high-risk essential thrombocythemia.
18080748	The potential risk of neoadjuvant chemotherapy in breast cancer patients--results from a prospective randomized trial of the Austrian Breast and Colorectal Cancer Study Group (ABCSG-07).
26970533	Rituximab extended schedule or retreatment trial for low tumour burden non-follicular indolent B-cell non-Hodgkin lymphomas: Eastern Cooperative Oncology Group Protocol E4402.
12149301	Phase III study of gemcitabine in combination with fluorouracil versus gemcitabine alone in patients with advanced pancreatic carcinoma: Eastern Cooperative Oncology Group Trial E2297.
9440749	Intermediate-dose intravenous methotrexate with intravenous mercaptopurine is superior to repetitive low-dose oral methotrexate with intravenous mercaptopurine for children with lower-risk B-lineage acute lymphoblastic leukemia: a Pediatric Oncology Group phase III trial.
10561297	Initial paclitaxel improves outcome compared with CMFP combination chemotherapy as front-line therapy in untreated metastatic breast cancer.
16123223	Frontline therapy with rituximab added to the combination of cyclophosphamide, doxorubicin, vincristine, and prednisone (CHOP) significantly improves the outcome for patients with advanced-stage follicular lymphoma compared with therapy with CHOP alone: results of a prospective randomized study of the German Low-Grade Lymphoma Study Group.

© 2019 Rubinstein SM et al. *JAMA Network Open.*

23228172	Abiraterone in metastatic prostate cancer without previous chemotherapy.
26500139	Lenalidomide with or without erythropoietin in transfusion-dependent erythropoiesis-stimulating agent-refractory lower-risk MDS without 5q deletion.
16230678	Randomized comparison of weekly cisplatin or protracted venous infusion of fluorouracil in combination with pelvic radiation in advanced cervix cancer: a gynecologic oncology group study.
23616624	Bortezomib consolidation after autologous stem cell transplantation in multiple myeloma: a Nordic Myeloma Study Group randomized phase 3 trial.
24038026	Efficacy, safety and survival with ruxolitinib in patients with myelofibrosis: results of a median 2-year follow-up of COMFORT-I.
24366758	Addition of docetaxel to S-1 without platinum prolongs survival of patients with advanced gastric cancer: a randomized study (START).
2230868	High- versus standard-dose megestrol acetate in women with advanced breast cancer: a phase III trial of the Piedmont Oncology Association.
8315422	Dose-response relationship of epirubicin-based first-line chemotherapy for advanced breast cancer: a prospective randomized trial.
11013280	Phase III comparative study of high-dose cisplatin versus a combination of paclitaxel and cisplatin in patients with advanced non-small-cell lung cancer.
19536890	Integrated data from 2 randomized, double-blind, placebo-controlled, phase 3 trials of active cellular immunotherapy with sipuleucel-T in advanced prostate cancer.
19536890	Integrated data from 2 randomized, double-blind, placebo-controlled, phase 3 trials of active cellular immunotherapy with sipuleucel-T in advanced prostate cancer.
20516439	Phase III study of the value of thalidomide added to melphalan plus prednisone in elderly patients with newly diagnosed multiple myeloma: the HOVON 49 Study.
15591112	Early consolidation by myeloablative radiochemotherapy followed by autologous stem cell transplantation in first remission significantly prolongs progression-free survival in mantle-cell lymphoma: results of a prospective randomized trial of the European MCL Network.
28677826	Elotuzumab plus lenalidomide/dexamethasone for relapsed or refractory multiple myeloma: ELOQUENT-2 follow-up and post-hoc analyses on progression-free survival and tumour growth.
7989940	Placebo-controlled randomized trial of infusional fluorouracil during standard radiotherapy in locally advanced head and neck cancer.
2184135	Adjuvant treatment of operable stomach cancer with polyadenylic.polyuridylic acid in addition to chemotherapeutic agents: a preliminary report.
16376489	French multicenter phase III randomized study testing concurrent twice-a-day radiotherapy and cisplatin/5-fluorouracil chemotherapy (BiRCF) in unresectable pharyngeal carcinoma: Results at 2 years (FNCLCC-GORTEC).
15812080	Overall survival after concurrent cisplatin-radiotherapy compared with radiotherapy alone in locoregionally advanced nasopharyngeal carcinoma.
15111618	High-dose chemotherapy with autologous hematopoietic stem-cell support compared with standard-dose chemotherapy in breast cancer patients with 10 or more positive lymph nodes: first results of a randomized trial.
8370422	Multiple myeloma treated with mitoxantrone in combination with vincristine and prednisolone (NOP regimen) versus melphalan and prednisolone: a phase III study. Nordic Myeloma Study Group (NMSG).
29427355	Rituximab, cladribine, and cyclophosphamide (RCC) induction with rituximab maintenance in chronic lymphocytic leukemia: PALG - CLL4 (ML21283) trial.
19195817	Comparison of 6 cycles versus 4 cycles of neoadjuvant epirubicin plus docetaxel chemotherapy in stages II and III breast cancer.

26751236	Cytoreductive surgery and intraperitoneal chemotherapy versus systemic chemotherapy for colorectal peritoneal metastases: A randomised trial.
7931476	First isolated locoregional recurrence following mastectomy for breast cancer: results of a phase III multicenter study comparing systemic treatment with observation after excision and radiation. Swiss Group for Clinical Cancer Research.
11181662	Phase III trial of standard-dose intravenous cisplatin plus paclitaxel versus moderately high-dose carboplatin followed by intravenous paclitaxel and intraperitoneal cisplatin in small-volume stage III ovarian carcinoma: an intergroup study of the Gynecologic Oncology Group, Southwestern Oncology Group, and Eastern Cooperative Oncology Group.
12065558	Superior survival with capecitabine plus docetaxel combination therapy in anthracycline-pretreated patients with advanced breast cancer: phase III trial results.
1991261	Combination hormonal therapy with tamoxifen plus fluoxymesterone versus tamoxifen alone in postmenopausal women with metastatic breast cancer. An updated analysis.
8960474	Intraperitoneal cisplatin plus intravenous cyclophosphamide versus intravenous cisplatin plus intravenous cyclophosphamide for stage III ovarian cancer.
27690294	Phase III randomized trial of autologous cytokine-induced killer cell immunotherapy for newly diagnosed glioblastoma in Korea.
23235801	Phase III study of ACVBP versus ACVBP plus rituximab for patients with localized low-risk diffuse large B-cell lymphoma (LNH03-1B).
16275936	High-dose therapy and autologous blood stem-cell transplantation compared with conventional treatment in myeloma patients aged 55 to 65 years: long-term results of a randomized control trial from the Group Myelome-Autogreffe.
17614302	Trastuzumab plus vinorelbine or taxane chemotherapy for HER2-overexpressing metastatic breast cancer: the trastuzumab and vinorelbine or taxane study.
8649495	A prospective, randomized trial of autologous bone marrow transplantation and chemotherapy in multiple myeloma. Intergroupe Fran√ßais du My√©lome.
10735496	Interferon-gamma in the first-line therapy of ovarian cancer: a randomized phase III trial.
15961764	Randomized phase II/III trial of interferon Alfa-2a with and without 13-cis-retinoic acid in patients with progressive metastatic renal cell Carcinoma: the European Organisation for Research and Treatment of Cancer Genito-Urinary Tract Cancer Group (EORTC 30951).
28005247	Efficacy and safety of low-dose capecitabine plus docetaxel versus single-agent docetaxel in patients with anthracycline-pretreated HER2-negative metastatic breast cancer: results from the randomized phase III JO21095 trial.
24150216	Chemomodulation of sequential high-dose cytarabine by fludarabine in relapsed or refractory acute myeloid leukemia: a randomized trial of the AMLCG.
28914260	High-dose methotrexate therapy significantly improved survival of adult acute lymphoblastic leukemia: a phase III study by JALSG.
6688538	A comparison of cyclophosphamide, adriamycin, and 5-fluorouracil (CAF) and cyclophosphamide, methotrexate, 5-fluorouracil, vincristine, and prednisone (CMFVP) in patients with advanced breast cancer.
16325695	Comparison of rapidly cycled tandem high-dose chemotherapy plus peripheral-blood stem-cell support versus dose-dense conventional chemotherapy for adjuvant treatment of high-risk breast cancer: results of a multicentre phase III trial.
21896539	Capecitabine versus 5-fluorouracil/folinic acid as adjuvant therapy for stage III colon cancer: final results from the X-ACT trial with analysis by age and preliminary evidence of a pharmacodynamic marker of efficacy.
991103	Combination chemotherapy for metastatic breast carcinoma. Prospective comparison of multiple drug therapy with L-phenylalanine mustard.

2503724	A controlled trial of leuprolide with and without flutamide in prostatic carcinoma.
1988573	A comparison of induction and maintenance therapy for acute nonlymphocytic leukemia in childhood: results of a Pediatric Oncology Group study.
17765354	Docetaxel versus docetaxel plus gemcitabine as front-line treatment of patients with advanced non-small cell lung cancer: a randomized, multicenter phase III trial.
23139262	A randomized clinical trial of adjuvant chemotherapy with doxorubicin, ifosfamide, and cisplatin followed by radiotherapy versus radiotherapy alone in patients with localized uterine sarcomas (SARCGYN study). A study of the French Sarcoma Group.
25102853	GEM2005 trial update comparing VMP/VTP as induction in elderly multiple myeloma patients: do we still need alkylators?
2901037	Combination adjuvant chemotherapy for node-positive breast cancer. Inadequacy of a single perioperative cycle.
14673047	Surgery plus chemotherapy compared with surgery alone for localized squamous cell carcinoma of the thoracic esophagus: a Japan Clinical Oncology Group Study--JCOG9204.
9739438	Randomized trial comparing monthly low-dose leucovorin and fluorouracil bolus with weekly high-dose 48-hour continuous-infusion fluorouracil for advanced colorectal cancer: a Spanish Cooperative Group for Gastrointestinal Tumor Therapy (TTD) study.
15598977	Angiogenesis inhibitor IM862 is ineffective against AIDS-Kaposi's sarcoma in a phase III trial, but demonstrates sustained, potent effect of highly active antiretroviral therapy: from the AIDS Malignancy Consortium and IM862 Study Team.
15860487	Randomized phase III trial comparing cisplatin-etoposide to carboplatin-paclitaxel in advanced or metastatic non-small cell lung cancer.
17822748	A gynecologic oncology group randomized phase III trial of whole abdominal irradiation (WAI) vs. cisplatin-ifosfamide and mesna (CIM) as post-surgical therapy in stage I-IV carcinosarcoma (CS) of the uterus.
16878325	Combined cyclophosphamide, vincristine, doxorubicin, and prednisone (CHOP) improves response rates but not survival and has lower hematologic toxicity compared with combined mitoxantrone, chlorambucil, and prednisone (MCP) in follicular and mantle cell lymphomas: results of a prospective randomized trial of the German Low-Grade Lymphoma Study Group.
7946592	Comparison of chemotherapy with or without medroxyprogesterone acetate for advanced or recurrent breast cancer.
15367414	Phase III trial of liposomal doxorubicin and cyclophosphamide compared with epirubicin and cyclophosphamide as first-line therapy for metastatic breast cancer.
18029971	Phase III trial of adjuvant 5-fluorouracil and adriamycin versus 5-fluorouracil, adriamycin, and polyadenylic-polyuridylic acid (poly A:U) for locally advanced gastric cancer after curative surgery: final results of 15-year follow-up.
30315239	Updated results from the phase 3 HELIOS study of ibrutinib, bendamustine, and rituximab in relapsed chronic lymphocytic leukemia/small lymphocytic lymphoma.
25605862	Phase III open-label randomized study of eribulin mesylate versus capecitabine in patients with locally advanced or metastatic breast cancer previously treated with an anthracycline and a taxane.
3897470	Phase III study of BCOP v CHOP in unfavorable categories of malignant lymphoma: a Southeastern Cancer Study Group trial.
12086759	Aggressive conventional chemotherapy compared with high-dose chemotherapy with autologous haemopoietic stem-cell transplantation for relapsed chemosensitive Hodgkin's disease: a randomised trial.
18467316	Phase III trial comparing intensive induction chemoradiotherapy (60 Gy, infusional 5-FU and intermittent cisplatin) followed by maintenance gemcitabine

© 2019 Rubinstein SM et al. *JAMA Network Open.*

	with gemcitabine alone for locally advanced unresectable pancreatic cancer. Definitive results of the 2000-01 FFCD/SFRO study.
12488405	Randomized adjuvant trial of tamoxifen and goserelin versus cyclophosphamide, methotrexate, and fluorouracil: evidence for the superiority of treatment with endocrine blockade in premenopausal patients with hormone-responsive breast cancer--Austrian Breast and Colorectal Cancer Study Group Trial 5.
16500914	Randomised Phase III study of biweekly 24-h infusion of high-dose 5FU with folinic acid and oxaliplatin versus monthly plus 5-FU/folinic acid in first-line treatment of advanced colorectal cancer.
18691879	Carboplatin and paclitaxel versus cisplatin, paclitaxel and doxorubicin for first-line chemotherapy of advanced ovarian cancer: a Hellenic Cooperative Oncology Group (HeCOG) study.
30833647	Phase III randomised trial comparing 6 vs. 12-month of capecitabine as adjuvant chemotherapy for patients with stage III colon cancer: final results of the JFMC37-0801 study.
363253	Adriamycin versus methotrexate in five-drug combination chemotherapy for advanced breast cancer: a randomized trial.
16110015	Randomized phase III study of docetaxel compared with paclitaxel in metastatic breast cancer.
3903501	A randomized trial of chemotherapy and hormonal therapy in advanced breast cancer.
23661293	Long-term follow-up of a phase III study comparing radiotherapy with or without weekly oxaliplatin for locoregionally advanced nasopharyngeal carcinoma.
8168039	Randomized trial of cyclophosphamide, methotrexate, and 5-fluorouracil with or without estrogenic recruitment in women with metastatic breast cancer.
2066763	Cisplatin and etoposide as first-line chemotherapy for metastatic breast carcinoma: a prospective randomized trial of the Italian Oncology Group for Clinical Research.
2644533	Prolonged disease-free survival after one course of perioperative adjuvant chemotherapy for node-negative breast cancer.
27614621	A phase III multicenter, randomized, controlled study of combined androgen blockade with versus without zoledronic acid in prostate cancer patients with metastatic bone disease: results of the ZAPCA trial.
2433409	Long-term results of combined chemotherapy-radiotherapy approach in Hodgkin's disease: superiority of ABVD plus radiotherapy versus MOPP plus radiotherapy.
9519355	Maximal androgen blockade: final analysis of EORTC phase III trial 30853. EORTC Genito-Urinary Tract Cancer Cooperative Group and the EORTC Data Center.
11474254	CMF (cyclophosphamide, methotrexate, 5-fluorouracil) versus cnf (cyclophosphamide, mitoxantrone, 5-fluorouracil) as adjuvant chemotherapy for stage II lymph-node positive breast cancer: a phase III randomized multicenter study.
28072706	A randomized, multicenter, phase III study of gemcitabine combined with capecitabine versus gemcitabine alone as first-line chemotherapy for advanced pancreatic cancer in South Korea.
10963641	Disease-free survival advantage of adjuvant cyclophosphamide, methotrexate, and fluorouracil in patients with node-negative, rapidly proliferating breast cancer: a randomized multicenter study.
2820289	Superiority of alternating non-cross-resistant chemotherapy in extensive small cell lung cancer. A multicenter, randomized clinical trial by the National Cancer Institute of Canada.
6145832	Treatment of disseminated breast cancer with tamoxifen, aminoglutethimide, hydrocortisone, and danazol, used in combination or sequentially.

10707786	A randomized British National Lymphoma Investigation trial of CHOP vs. a weekly multi-agent regimen (PACEBOM) in patients with histologically aggressive non-Hodgkin's lymphoma.
1105174	1-Phenylalanine mustard (L-PAM) in the management of primary breast cancer. A report of early findings.
3219274	Prednisolone improves the response to primary endocrine treatment for advanced breast cancer.
9309100	Adjuvant radiotherapy and chemotherapy in node-positive premenopausal women with breast cancer.
1105174	1-Phenylalanine mustard (L-PAM) in the management of primary breast cancer. A report of early findings.
23223332	Docetaxel-cisplatin might be superior to docetaxel-capecitabine in the first-line treatment of metastatic triple-negative breast cancer.
6105336	Adjuvant treatment with polyadenylic-polyuridylic acid (Polya.Polyu) in operable breast cancer.
17690257	Extended follow-up of a phase 3 trial in relapsed multiple myeloma: final time-to-event results of the APEX trial.
25676423	A randomised comparison of the novel nucleoside analogue sapacitabine with low-dose cytarabine in older patients with acute myeloid leukaemia.
6252466	Streptozocin alone compared with streptozocin plus fluorouracil in the treatment of advanced islet-cell carcinoma.
7718316	A randomised study to compare the effect of the luteinising hormone releasing hormone (LHRH) analogue goserelin with or without tamoxifen in pre- and perimenopausal patients with advanced breast cancer.
29165021	Two years of tamoxifen or no adjuvant systemic therapy for patients with high-risk breast cancer: long-term follow-up of the Copenhagen breast cancer trial.
19567453	Rituximab versus observation after high-dose consolidative first-line chemotherapy with autologous stem-cell transplantation in patients with poor-risk diffuse large B-cell lymphoma.
8996149	Controlled trial of fluorouracil and low-dose leucovorin given for 6 months as postoperative adjuvant therapy for colon cancer.
7910230	Efficacy of immunochemotherapy as adjuvant treatment after curative resection of gastric cancer. Study Group of Immunochemotherapy with PSK for Gastric Cancer.
7880604	Weekly doxorubicin with or without high-dose medroxyprogesterone acetate in hormone-resistant advanced breast cancer. A randomised study. The Norwegian Breast Cancer Group.
20644101	Bortezomib plus dexamethasone induction improves outcome of patients with t(4;14) myeloma but not outcome of patients with del(17p).
2167953	A randomized study comparing cisplatin or carboplatin with etoposide in patients with advanced non-small-cell lung cancer: European Organization for Research and Treatment of Cancer Protocol 07861.
6132179	Controlled trial of adjuvant chemotherapy with melphalan for breast cancer.
21502557	International phase III trial assessing neoadjuvant cisplatin, methotrexate, and vinblastine chemotherapy for muscle-invasive bladder cancer: long-term results of the BA06 30894 trial.
8443401	Second and third line hormonotherapy in advanced post-menopausal breast cancer: a multicenter randomized trial comparing medroxyprogesterone acetate with aminoglutethimide in patients who have become resistant to tamoxifen.
22184384	Phase III trial comparing capecitabine plus cisplatin versus capecitabine plus cisplatin with concurrent capecitabine radiotherapy in completely resected gastric cancer with D2 lymph node dissection: the ARTIST trial.
348293	A randomized comparative trial of adriamycin versus methotrexate in combination drug therapy.

© 2019 Rubinstein SM et al. *JAMA Network Open.*

1832904	"Classical" CMF versus a 3-weekly intravenous CMF schedule in postmenopausal patients with advanced breast cancer. An EORTC Breast Cancer Co-operative Group Phase III Trial (10808).
329975	A comparison of cyclophosphamide, adriamycin, 5-fluorouracil (CAF) and cyclophosphamide, methotrexate, 5-fluorouracil, vincristine, prednisone (CMFVP) in patients with metastatic breast cancer: a Southeastern Cancer Study Group project.

© 2019 Rubinstein SM et al. *JAMA Network Open.*
